# Promoted Osprey Optimizer: a solution for ORPD problem with electric vehicle penetration

**DOI:** 10.1038/s41598-024-79185-6

**Published:** 2024-11-14

**Authors:** Ziang Liu, Xiangzhou Jian, Touseef Sadiq, Zaffar Ahmed Shaikh, Osama Alfarraj, Fahad Alblehai, Amr Tolba

**Affiliations:** 1 Department of Electrical and Computer Engineering, Carnegie Mellon University, Pennsylvania, 15213 USA; 2 Department of Mechanical Engineering, Columbia University, New York, 10027 USA; 3 Centre for Artificial Intelligence Research (CAIR), Department of Information and Communication Technology, University of Agder, Jon Lilletuns vei 9, Grimstad, Norway; 4 Department of Computer Science and Information Technology, Benazir Bhutto Shaheed University Lyari, Karachi, 75660 Pakistan; 5 Computer Science Department, Community College, King Saud University, Riyadh, 11437 Saudi Arabia; 6 School of Engineering, École Polytechnique Fédérale de Lausanne, 1015 Lausanne, Switzerland

**Keywords:** Optimal reactive power dispatch, ORPD Electric Vehicles, Promoted Osprey Optimizer, Metaheuristic optimization, Power System Operation, Energy science and technology, Engineering

## Abstract

This paper proposes a new optimization technique to make an integration between the Optimal Reactive Power Dispatch (ORPD) problem and Electric Vehicles (EV). Here, a modified metaheuristic algorithm, called the Promoted Osprey Optimizer (POO) is used for this purpose. Inspired by the hunting behavior of ospreys, a predatory bird species, the POO algorithm employs various strategies like diving, soaring, and gliding to effectively explore the search space and avoid local optima. To evaluate its performance, the POO-based model has been applied to the IEEE 118-bus and IEEE 57-bus systems, considering different scenarios of EV penetration. The experimental findings demonstrate that the POO algorithm can effectively optimize the reactive power dispatch problem with EV integration, achieving significant reductions in active power losses and voltage deviations toward several existing metaheuristic optimization techniques in different terms. The POO algorithm demonstrates a significant reduction in power loss, achieving up to 22.2% and 16.2% in the 57-bus and 118-bus systems, respectively. This improvement is accompanied by reductions in voltage deviation of up to 20.6% and 15.7%. In the 57-bus system, power loss is reduced from 2.35 MW to 1.93 MW, while voltage deviation decreases from 0.034 p.u. to 0.027 p.u. For the 118-bus system, power loss is lowered from 4.21 MW to 3.53 MW, and voltage deviation is reduced from 0.051 p.u. to 0.043 p.u. Furthermore, the POO algorithm surpasses other optimization methods in minimizing voltage deviation, achieving reductions of up to 0.056 p.u. in the 57-bus system and up to 0.163 p.u. in the 118-bus system. Consequently, the POO algorithm holds great potential as a valuable tool for power system operators and planners to optimize reactive power dispatch and enhance power system performance with EV integration.

## Introduction

The complexity of Optimal Reactive Power Dispatch (ORPD) is increasing due to the growing number of Electric Vehicles (EVs), which have a significant impact on the dynamics of the power grid. ORPD aims to find the best configuration for control variables while minimizing power losses, maintaining voltage profiles within predetermined limits, and ensuring system stability and power flow reliability. However, the rise in power demand caused by EV charging presents a challenge to maintaining an efficient ORPD^1^.

EVs can worsen voltage fluctuations and losses, as well as put additional stress on transmission and distribution networks. On the other hand, EVs can also function as distributed energy resources through bidirectional power flow, particularly with Vehicle-to-Grid (V2G) systems. This V2G interaction allows EV batteries to discharge back into the grid, providing support for grid services such as demand response and voltage regulation^2^.

However, integrating this capability requires careful coordination of EV charging schedules, considering the battery capacity and state of charge of the vehicles, to ensure optimal utilization without compromising the driving needs of users. Consequently, the ORPD problem must now incorporate these additional variables and strike a balance between the operational demands of the grid and the complexities arising from the decentralized and mobile nature of EVs, which act as both consumers and potential electricity suppliers^3^.

Indeed, EVs have a dual influence on the power grid, encompassing both adverse and beneficial effects. However, EVs can induce voltage fluctuations and losses, especially during the processes of charging and discharging. This occurs as they create imbalanced loads on the grid, resulting in voltage drops and increased power losses. Furthermore, EVs can impose additional strain on transmission and distribution networks, particularly during peak demand periods, which may lead to power outages and equipment malfunctions. Conversely, EVs also offer positive contributions, as they can act as distributed energy resources. Through bidirectional power flow in Vehicle-to-Grid (V2G) systems, EVs can return energy to the grid, facilitating V2G interactions. This capability allows EV batteries to discharge energy back into the grid, thereby supporting essential grid services such as demand response and voltage regulation. Ultimately, it causes EVs a significant asset for managing peak demand and ensuring grid stability.

The incorporation of Electric Vehicles (EVs) into the power system presents both challenges and opportunities for the Optimal Reactive Power Dispatch (ORPD) problem. EVs have the potential to increase power demand and impact the load profile, voltage profile, and power quality of the power system^4^. Additionally, EVs can serve as distributed energy resources, offering additional services like reactive power support, frequency regulation, and peak shaving through their batteries and bidirectional chargers^5^. However, effectively managing the integration of ORPD with EV necessitates careful consideration of various factors, including charging schedules, battery capacity, state of charge, and EV location. Furthermore, the ORPD problem becomes more complex and extensive with the inclusion of EVs, demanding efficient and robust optimization techniques for its resolution^6^.

Metaheuristic algorithms present a viable solution for addressing the complexities of the Optimized Reactive Power Dispatch (ORPD) problem, especially in the presence of Electric Vehicles (EVs). These algorithms offer a versatile framework for developing heuristic optimization algorithms, making them well-suited for tackling large and intricate optimization problems that traditional methods struggle to handle. When applied to ORPD in the context of EV integration, metaheuristics offer numerous advantages. Firstly, metaheuristics efficiently explore the solution space, enabling the identification of near-optimal control variable settings while considering factors, such as EV charging/discharging schedules, state of charge, and vehicle-to-grid (V2G) capabilities^7^.

This allows for effective management of EV-related variables. Secondly, metaheuristics excel at handling multiple objectives^8^. They can optimize power loss minimization, maintain voltage profiles within specific limits, and maximize the utilization of renewable energy sources, all while accounting for the optimal scheduling of EV charging to support grid stability^9^. This comprehensive approach ensures a balanced and efficient use of resources^10–14^. Thirdly, these algorithms demonstrate adaptability and flexibility in the face of the dynamic nature of the power grid with EVs^15^.

By incorporating real-time data and quickly adjusting to changes, they can effectively respond to varying EV charging demands and fluctuations in renewable energy generation levels^16^. This adaptability ensures the stability and reliability of the grid^17^. Lastly, metaheuristics offer computational efficiency despite the high-dimensionality of the ORPD problem. Their ability to avoid exhaustive enumeration and employ intelligent search strategies allows them to find good solutions with significantly reduced computational effort compared to exact methods. This efficiency makes them practical for solving real-world ORPD problems involving EV integration^18^.

In this field, Khan et al.^19^ conducted a research that was according to the Fractional Calculus with Particle Swarm Optimization Gravitational Search Algorithm (FPSOGSA). The purpose of the current algorithm was improving the traditional PSOGSA’s abilities of searching; therefore, it could solve the inclination problem of stagnation. The suggested algorithm was examined according to system of IEEE 30-bus in order to decline the losses of power and deviation of voltage then improve consistency of voltage. The approach based on scenario was utilized for generating several scenarios from load uncertainty, irradiation of solar, and speed of wind. The experimental outcomes certified the efficacy of the suggested algorithm to solve issue of ORPD without and with taking into account the system’s uncertainty. Additionally, the suggested model was better in comparison with some advanced approaches regarding decrease in losses of power, consistency improvement, and deviation of voltage.

Saddique et al.^20^ developed a novel Sine-Cosine Algorithm (SCA) for solving the issues of ORPD via taking independent and dependent control parameter restrictions into account. In accordance with power systems of 30-, 57-, and 14-, Sine-Cosine Algorithm was verified and examined. The outcomes illustrated a considerable enhancement in minimizing losses of power. Hence, by utilizing system of 14-bus, the power losses were diminished to the least from 4.78 to 0.04%. Whereas, it decreased from 3.4 to 0.4% utilizing system of 30-bus. Then, it was reduced from 1.99 to 0.9% by the utility of system of 57-bus. To investigate the efficacy of the study, it was contrasted with PSO (Particle Swarm Optimization), WOA (Whale Optimization Algorithm), and Differential Evolution (DE). The outcomes revealed that the suggested algorithm was computationally easy, efficacious, and strong while solving issues of ORPD.

Marcelino et al.^21^ addressed the issue of OARPD (Optimal Active–Reactive Power Dispatch) within microgrids utilizing EVs (Electric Vehicle). The enhanced systems of IEEE 118- and 57-bus exam scenarios were utilized that generators of thermoelectric were substituted by some generators that were renewable. Precisely, the EVs were combined with the grid under system of IEEE 118-bus. For solving the issue of OARDP, the enhancement and usage of Canonical Differential Evolutionary Particle Swarm Optimizer (C-DEEPSO) was recommended. To apply any modification within solution space, C-DEEPSO relied on local search. The outcomes illustrated that the enhanced model could reveal savings of production that served as controller of dispatch.

Chamba et al.^22^ presented the PSO’s application for gaining optimized reactive power dispatch. The efficacy of the method was illustrated via its high speed of processing. Moreover, the outcomes were gained via a thorough exploration for ORPD. Furthermore, the outcomes confirmed the efficacy of algorithm for optimization of performance index in various case studies that highlighted its capability for gaining ORPD. The present research represented a considerable accomplishment in optimizing system of power and prepared valuable device to manage and control those systems.

Shaheen et al.^23^ represented a new ETSO (Enhanced Transient Search Optimization) approach as the optimized solution of ORPD issue via combining EVs (Electric Vehicles). The suggested ETSO that was carried out as the optimized solution of ORPD issue minimized the loss of active power and deviation of voltage within buses of load. Suggested approach was examined and certified utilizing systems of IEEE118-, IEEE-57, and IEEE 30-bus. The outcomes demonstrated the efficacy and strength of the suggested optimizer to solve issues of ORPD via assessing and contrasting it to several well-founded optimizers using the identical data of system, limitations, and control parameters. The suggested algorithm’s experimental attributes and the accomplished outcomes resulted in an enhancement considering the efficacy of the systems of power.

Numerous research deficiencies exist within the current literature regarding reactive power dispatch issues associated with EV integration. These include the absence of robust optimization algorithms that were capable of addressing the complexity and non-linearity inherent in the problem, insufficient analysis of EV charging and discharging behaviors and their effects on the power grid, limited investigation into V2G functionalities that could enhance grid services, inadequate focus on power grid limitations such as transmission line capacities and voltage restrictions, a lack of thorough assessments of optimization algorithms, minimal consideration of uncertainties and fluctuations in EV charging and discharging patterns as well as renewable energy sources, and a pressing need for more realistic and applicable case studies to substantiate the efficacy of optimization algorithms. This study seeks to bridge these research gaps by advancing the development of effective optimization algorithms for reactive power dispatch challenges involving EV integration and enhancing the understanding of EVs’ influence on the power grid.

The objective of this paper is to propose a new metaheuristic optimization algorithm, named Promoted Osprey Optimizer (POO), for solving the ORPD problem with EV integration. The POO algorithm takes inspiration from the hunting behavior of ospreys, a type of bird of prey, and effectively explores the search space while avoiding local optima through various strategies like diving, soaring, and gliding. This paper makes a dual contribution, inlcluding (1) the development of a novel optimization framework that integrates Electric Vehicles (EVs) into the Optimal Reactive Power Dispatch (ORPD) problem, (2) the proposal of a robust and efficient Promoted Osprey Optimizer (POO) algorithm to solve the ORPD problem with EVs, (3) the demonstration of the effectiveness of the POO algorithm in reducing power loss and voltage deviation in power systems with EVs, and (4) the provision of a comprehensive analysis of the impact of EVs on power system operation and the benefits of using the proposed POO algorithm for ORPD with EVs. The POO algorithm can serve as a valuable tool for power system operators and planners in optimizing reactive power dispatch and enhancing power system performance with EV integration.

## Problem statement

### Fitness function

The Optimal Reactive Power Dispatch (ORPD) is a nonlinear optimality problem that aims to find the optimal configurations for voltage control devices and reactive power sources in a power system. Mathematically, the ORPD can be defined as follows:

1 $$\:\text{m}\text{i}\text{n}{F}_{i|i=\text{1,2}}(x,u)$$

Subject to:

2 $$\:g\left(x,u\right)=0,$$

3 $$\:h(x,u)\le\:0$$

The design variables are represented by the vector $\:u$, while the vector $\:x$ represents the dependent variables^24^. The objective function, $\:F(x,u)$, determines the desired outcomes of the ORPD problem, which can involve minimizing active power losses, voltage variation, or reactive power production costs. This function can be either single or multi-objective.

The constraints that need to be satisfied by the ORPD problem, such as power flow equations, voltage limitations, reactive power limits, and line flow restrictions, are expressed through the equality and inequality constraints $\:g\left(x,u\right)$ and $\:h(x,u)$, respectively. This study focuses on minimizing power loss and voltage deviation.

#### Voltage deviation reduction

The primary objective of voltage deviation is to enhance the voltage profile. The power system voltage profile pertains to the variation in voltage values at the load buses from their anticipated values. Typically, it is usual to put the desired voltage value at load buses as 1.0 per unit. The voltage profile measurement can be established by the following formula:

4 $$\:{F}_{1}=\text{min}\sum\:_{i=1}^{{M}_{L}}{\left({V}_{i}-{V}_{i}^{D}\right)}^{2}$$

where, $\:{M}_{L}$​ specifies the bus load numbers, and $\:{V}_{i}^{D}$​ and $\:{V}_{i}$ represent the desired voltage and the load voltage at $\:{i}^{th}$ bus.

#### Reducing loss of power

The primary objective is to decrease the quantity of active power loss. The total dissipation of active power in a power system is determined by the cumulative losses that occur across all branches^25^. The power loss in a branch is influenced by various factors, such as voltage magnitudes, conductance, and the voltage angle difference among connected buses. To minimize the active power loss, the following equation has been employed:

5 $$\:{F}_{2}=\text{m}\text{i}\text{n}\left({P}_{loss}\right)=\text{m}\text{i}\text{n}\sum\:_{k=1}^{{N}_{TL}}{G}_{k}\left({V}_{i}^{2}+{V}_{j}^{2}-2{V}_{i}{V}_{j}\text{cos}{\theta\:}_{ij}\right)$$

where, $\:{G}_{k}$ specifies the branch $\:k$ conductance that connects busses $\:i$ and $\:j$, $\:{P}_{loss}$ specifies the active power loss, $\:{N}_{TL}$ signifies the total number of transmission lines/branches, $\:{\theta\:}_{ij}$ represents the branches’ angle of admittance busses $\:i$ and $\:j$, and $\:{V}_{i}$​ and $\:{V}_{j}$ represent, in turn, the voltage scales of the bus $\:i$ and the bus $\:j$.

The ORPD’s determination has undergone a series of inequality and equality constraints, encompassing the physical and operational constraints of the power system. These constraints are expressed in the following.

#### Equality constraints

The constraints guarantee the preservation of power balance at every bus within the system, ensuring that the over-all inserted power at each bus is equivalent to the power flowing through the interconnected branches. The equality constraints are outlined as follows:

6 $$\:{P}_{gi}-{P}_{di}-{V}_{i}\sum\:_{j=1}^{{N}_{B}}{V}_{j}{G}_{k}\text{cos}{\theta\:}_{k}+{{V}_{j}B}_{k}\text{sin}{\theta\:}_{k}=0$$

7 $$\:{Q}_{gi}+{Q}_{ci}-{Q}_{di}-{V}_{i}\sum\:_{j=1}^{{N}_{B}}{V}_{j}{G}_{k}\text{s}\text{i}\text{n}{\theta\:}_{k}-{V}_{j}{B}_{k}\text{c}\text{o}\text{s}{\theta\:}_{k}=0$$

where, $\:i\in\:{N}_{L}$. $\:{B}_{k}$ specifies the susceptance during busses $\:i$ and $\:j$ in a power network. $\:{N}_{B}$ describes the total number of buses, $\:{N}_{L}$ specifies the total number of load buses within the network of power, and $\:{Q}_{ci}$ describes the reactive power compensation. $\:{Q}_{gi}$ and $\:{P}_{gi}$ state the reactive and active power generation in bus $\:i$. Similarly, $\:{Q}_{di}$ and $\:{P}_{di}$ represent, in turn, the reactive and active power demand with bus $\:i$.

#### Inequality constraints

This research uses inequality constraints with a variety of variables, such as transformer tap settings, branch power flow, voltage magnitudes, reactive power production, reactive power compensation, and the slack bus power. Moreover, the inequality restrictions are as follows:

8 $$\:{P}_{s}^{\text{m}\text{i}\text{n}}\le\:{P}_{s}\le\:{P}_{s}^{\text{m}\text{a}\text{x}}$$

9 $$\:{S}_{k}\le\:{S}_{k}^{\text{m}\text{a}\text{x}},k\in\:{N}_{l}$$

10 $$\:{V}_{i}^{\text{m}\text{i}\text{n}}\le\:{V}_{i}\le\:{V}_{i}^{\text{m}\text{a}\text{x}},i\in\:{N}_{B}$$

11 $$\:{Q}_{ci}^{\text{m}\text{i}\text{n}}\le\:{Q}_{ci}\le\:{Q}_{ci}^{\text{m}\text{a}\text{x}},i\in\:{N}_{c}$$

12 $$\:{T}_{m}^{\text{m}\text{i}\text{n}}\le\:{T}_{m}\le\:{T}_{m}^{\text{m}\text{a}\text{x}},m\in\:{N}_{t}$$

13 $$\:{Q}_{gi}^{\text{m}\text{i}\text{n}}\le\:{Q}_{gi}\le\:{Q}_{gi}^{\text{m}\text{a}\text{x}},i\in\:{N}_{pv}$$

where, $\:{S}_{k}$ represents the complex power over branch $\:k$, $\:{N}_{pv}$ describes the photovoltaic buses value, $\:{T}_{m}$ illustrates the transformer tapping alteration, $\:{N}_{l}$ defines the number of branches, $\:{N}_{t}$ describes the quantity of transformer tapings, $\:{P}_{s}$ signifies the real power of the slack bus, and $\:{N}_{c}$ signifies the number of buses with reactive power compensation.

## Osprey optimizer

Within the current stage, the suggested algorithm and the statistical modeling of it have been explained.

### Inspiration

This animal, called river, sea, and fish hawk, is a bird that eats fish and lives at night that is of different kind. The length, weight, and wingspan of this animal are between, in turn, 50 and 66 cm, 0.9 and 2.1 kg, and 127 and 180 cm. The exterior features of the animal have been explained in the following^26^. Considering the parts in the upside, they are bright brown; however, the breast has been witnessed to be white, and there are some brown dots on it^27^. The part in the downside is completely white. Its head has been seen to be white that has a black cover from its eyes to the neck.

The animal’s irises are something between brown and golden, and its nictitating membrane has been witnessed to be light blue. Its beak has been considered to be black and the color of its feet is black, and its talons are black. The tail and wings of this animal are, in turn, short and narrow-long.

The present animal is a one whose diet is mostly fish. The animal often hunts alive fish with weight of 300 to 150 g and length of 35 –25 cm. But it has the ability to hunt fish with the weight of 2 kg to 50 g. these animal possess a great vision that can diagnose the location of other fish below water even when they fly 10–40 m above the water. After that, it goes to the fish and dives into the water for hunting the fish. When the animal hunts the target, it transfers the target to a close rock and commences to consume the quarry.

The technique of this animal for catching and carrying to an appropriate location for eating have been considered to be intelligent manner that are foundation of developing a novel optimizer. For this reason, the statistical modeling of the animals’ intelligent manners has been utilized while designing the suggested algorithm that has been thoroughly explained subsequently.

### Statistical modeling

Here, the act of initializing the current algorithm has been explained. After that, the procedure of enhancing the animals’ location has been explained that are within two stages of exploitation and exploration on the basis of imitating the natural manners of the current animals.

#### Initialization

The suggested algorithm is a method based on the population that has the capability to supply an appropriate solution on the basis of power of search within the search space by a procedure that is based on iteration. The values of problem parameters have been determined by all animals in accordance with their locations within the solution space. So, all animals have been considered to be individual solutions for the problem that have been statistically developed by the use of a vector. These animals make the population of the intended algorithm that can be simulated by the use of matrix below. At the start stage of implementing OOA, these animals’ locations within the solution space have been stochastically initialized by the use of equation below:

14 $$\:Y = {\left[ {\begin{array}{*{20}{c}}{{Y_1}}\\{\: \vdots }\\{\:\begin{array}{*{20}{c}}{{Y_j}}\\{\:\begin{array}{*{20}{c}}\vdots \\{\:{Y_N}}\end{array}}\end{array}}\end{array}} \right]_{N \times \:k}} = {\left[ {\begin{array}{*{20}{c}}{{Y_{{\rm{1,1}}}}}&{\: \cdots \:}&{\:\begin{array}{*{20}{c}}{{Y_{1,i}}}&{\: \cdots \:}&{\:{Y_{1,k}}}\end{array}}\\{\: \vdots }&{\: \ddots \:}&{\:\begin{array}{*{20}{c}}\vdots &{\:\:\:\:\:\: \vdots }&{\:\:\:\: \vdots \:\:}\end{array}}\\{\:\begin{array}{*{20}{c}}{{Y_{j,1}}}\\\vdots \\{\:{Y_{N,1}}}\end{array}}&{\:\begin{array}{*{20}{c}}{ \cdots \:}\\{\: \vdots }\\{\: \cdots \:}\end{array}}&{\:\begin{array}{*{20}{c}}{\begin{array}{*{20}{c}}{{Y_{j,i}}}\\{\: \vdots }\\{\:{Y_{N,i}}}\end{array}}&{\:\begin{array}{*{20}{c}}{ \cdots \:\:\:}\\{\: \ddots \:\:\:\:}\\{\: \cdots \:}\end{array}\:}&{\:\begin{array}{*{20}{c}}{{Y_{j,k}}}\\{\: \vdots }\\{\:{Y_{N,k}}}\end{array}}\end{array}}\end{array}} \right]_{N \times \:k}},$$

15 $$\:{Y}_{j,i}=l{b}_{i}+{r}_{j,i}\times\:\left(u{b}_{i}-l{b}_{i}\right),\:j=1,\:2,\:.\:.\:.\:,\:k,$$

here, matrix population of the animals’ situations has been illustrated by $\:Y$. The animal $\:j$, known as individual solution, has been depicted by $\:{Y}_{j}$, the dimension $\:i$ has been signified by $\:{Y}_{j,i}$, the quantity of the animal has been indicated by $\:N$, $\:k$ displays the quantity of problem parameters, and the stochastic quantities within the range 0 and 1 have been demonstrated by $\:{r}_{j,i}$. Moreover, the upper and lower boundaries of the problem parameter $\:i$ have been, in turn, illustrated by $\:u{b}_{i}$ and $\:l{b}_{i}$.

It is possible to assess the performance index because all animals have been considered individual solution. The values that have been assessed for the performance index of the issue might be illustrated by the use of a vector.

16 $$\:G = {\left[ {\begin{array}{*{20}{c}}{{G_1}\:}\\\vdots \\{\:\begin{array}{*{20}{c}}{{G_j}}\\{\:\begin{array}{*{20}{c}}\vdots \\{\:{G_N}}\end{array}}\end{array}}\end{array}} \right]_{N \times \:1}} = {\left[ {\begin{array}{*{20}{c}}{G\left( {{Y_1}} \right)}\\{\: \vdots }\\{\:\begin{array}{*{20}{c}}{G\left( {{Y_j}} \right)}\\{\: \vdots }\\{\:G\left( {{Y_N}} \right)}\end{array}}\end{array}} \right]_{N \times \:1}},$$

where, the values of performance index’s vector have been illustrated by $\:G$; in addition, the gained performance index value of the animal $\:j$ has been demonstrated by $\:{G}_{J}$.

The assessed values of the performance index have been found to be the key criteria to assess the individual solutions’ quality. Hence, the finest value that has been gained for the performance index displays the finest individual solution; moreover, the most terrible value that has been gained for the performance index displays the most terrible individual solution. Taking into account the location of these animals within the solution space has been upgraded within all iterations, the finest individual solution has to be upgraded as well.

#### Stage 1: recognition of location and chasing the target (global search)

These animals are huge predators and can recognize the position of target underwater because of their great vision ability. Once these animals identify the location of the targets, they go underwater and hunt it. The initial stage of update of population within the current algorithm has been simulated on the basis of natural manners of these animals. Simulating the animals’ attack considerably alters the locations of the animals within the solution space that raises the algorithm’s ability of global search to identify optimum region and evade local optimum.

Within design of the Osprey Optimization Algorithm, the location of the animals within the solution space that enjoy a superior value of performance index is determined by the target. The targets’ set for the individuals has been signified by the use of equation below:

17 $$\:G{p}_{j}=\left[{Y}_{z}|z\in\:\left\{1,\:2,\:.\:.\:.\:,\:N\right\}\wedge\:{G}_{z}<{G}_{j}\right]\cup\:\left[{Y}_{best}\right]$$,

here, the location of set of targets for the animal $\:j$ has been specified by $\:G{p}_{j}$; additionally, the finest individual solution has been illustrated by $\:{Y}_{best}$.

The individuals stochastically identify the location of a target and attack it. On the basis of the imitation of the animal’s motion to the target, the novel location of the animal has been computed by the use of Eq. (18). Once the novel location enhances the performance index, it substitutes the prior location of the animal in accordance with Eq. (19).

18a $$\:{Y}_{j,i}^{P1}={Y}_{j,i}+{r}_{j,i}\times\:\left(H{G}_{j,i}-{I}_{j,i}\right)\times\:{Y}_{j,i}$$,

18b $$\:{Y}_{j,i}^{P1}=\left\{\begin{array}{c}{Y}_{j,i}^{P1},\:l{b}_{i}\le\:{Y}_{j,i}^{P1}\le\:u{b}_{i};\\\:l{b}_{i},\:{Y}_{j,i}^{P1}<l{b}_{i};\:\:\:\:\:\:\:\:\:\:\:\:\:\:\:\:\\\:u{b}_{i},\:{Y}_{j,i}^{P1}>u{b}_{i,}\:\:\:\:\:\:\:\:\:\:\:\:\:\:\end{array}\right.$$

19 $$\:{Y}_{j}\left\{\begin{array}{c}{Y}_{j}^{p1},\:{G}_{j}^{P1}<{G}_{j}\:\\\:{Y}_{j},\:else,\:\:\:\:\:\:\:\:\:\:\:\:\:\end{array}\right.$$

where, the novel location of the animal $\:j$ has been indicated by $\:{Y}_{j}^{P1}$ in accordance with the initial stage of the algorithm, the dimension $\:i$ has been demonstrated by $\:{Y}_{j,i}^{P1}$, the value of performance index has been specified by $\:{G}_{j}^{P1}$, the chosen target for the animal $\:j$ has been depicted by $\:H{G}_{j}$, the dimension $\:i$ has been signified by $\:H{G}_{j,i}$, $\:{r}_{j,i}$ is a stochastic quantity between 1 and 0; finally, $\:{I}_{j,i}$ is a random quantity within the range^1,2^.

#### Stage 2: moving the target to an appropriate place (local search)

Once, the individuals hunt a target, they move it to an appropriate location to consume it in order to ensure his safety. The subsequent stage of population update has been simulated in accordance with the imitation of the animal’s natural manner. The simulation of moving the target to an appropriate place makes the location of the animal experience some alteration within the solution space. Moreover, this leads to a raise in the algorithm’s local search ability and convergence to finer solutions.

Within the design of the OOA, a novel stochastic location has been computed utilizing Eq. (20), known as appropriate place to consume the target, for simulating the animal’s natural manner. After that, once objective function value has been enhanced, it gets substituted with the prior location of the animal in accordance with Eq. (21).

20a $$\:{Y}_{j,i}^{p2}={Y}_{j,i}+\frac{l{b}_{i}+r\times\:(u{b}_{i}-l{b}_{i})}{t},\:j=1,\:2,\:.\:.\:.\:,\:N,\:i=1,\:2,\:.\:.\:.\:,\:k,\:t=1,\:2,\:.\:.\:.\:,\:T$$,

20b $$\:{Y}_{j,i}^{p2}=\left\{\begin{array}{c}{Y}_{j,i}^{p2},\:l{b}_{i}\le\:{Y}_{j,i}^{p2}\le\:u{b}_{i};\\\:l{b}_{i},\:{Y}_{j,i}^{p2}<l{b}_{i};\:\:\:\:\:\:\:\:\:\:\:\:\:\:\\\:u{b}_{i},\:{Y}_{j,i}^{p2}>u{b}_{i},\:\:\:\:\:\:\:\:\:\:\:\:\:\end{array}\right.$$

21 $$\:{Y}_{j}=\left\{\begin{array}{c}{Y}_{j}^{P2},\:{G}_{j}^{P2}<{G}_{j};\\\:{Y}_{j},\:else,\:\:\:\:\:\:\:\:\:\:\:\:\:\end{array}\right.$$

here, the novel location of the animal $\:j$ has been illustrated by $\:{Y}_{j}^{p2}$ in accordance with the second stage of the algorithm, the dimension $\:i$ has been demonstrated by $\:{Y}_{j,i}^{p2}$, the value of performance index has been depicted by $\:{G}_{j}^{P2}$, the stochastic quantities that are between 1 and 0 have been illustrated by $\:{r}_{j,i}$, and the algorithm’s quantity of iterations has been displayed by $\:t$; in addition, the entire quantity of iteration has been signified by $\:T$.

The suggested algorithm is a method based on iteration. The initial iteration of the algorithm gets accomplished via upgrading locations of all animals in accordance with the second and first stage. After that, the finest individual solution gets upgraded in accordance with contrasting the values of performance index. Next, the OOA commences the subsequent iteration with novel locations of the animals. Moreover, the upgrade procedure of the algorithm keeps going till the final iteration on the basis of Eq. (17) to Eq. (21). Eventually, once the algorithm has been thoroughly implemented, the algorithm represents the finest individual as a solution.

### Promoted Osprey Optimizer

The following section presents a revised edition of the osprey optimizer, which is modified based on chaos map and opposition-based learning techniques. By employing opposition-based learning, the initial population becomes more widely distributed, while the incorporation of a chaotic map ensures a balance between exploitation and exploration factors. This modification aims to improve the convergence rate and modify the local optimum.

#### Opposition-based learning

The notion of Opposition-Based Learning (OBL) was initially introduced by Tizhoosh^28^ as a means of adapting optimization. This approach is widely recognized as a robust mathematical technique in the metaheuristic algorithms^29^. The initialization in this state commences with a random value. It is possible for the optimization process to commence with an initial value that is not the optimal solution, or in the most unfavorable scenario, and it may even begin in the opposite direction of the optimal solution.

Consequently, this leads to a prolonged duration for the search procedure compared to the standard situation. The Opposite-Based Learning (OBL) algorithm generates the opposite position alongside the original location within the initial population as follows:

22 $$\:{Y}_{j,i}^{op}={Y}_{j,i}^{max}+{Y}_{j,i}^{min}-{Y}_{j,i}$$

where, $\:{Y}_{j,i}^{op}$ specifies the opposite position of $\:{Y}_{j,i}$, and $\:{Y}_{j,i}^{min}$ and $\:{Y}_{j,i}^{max}$ represent minimum and the maximum limitations, respectively.

The alternative stance offers a greater array of possibilities for discovering the optimal solution. The measurement of $\:{Y}_{j,i}^{op}$ is conducted through the objective function. Consequently, if $\:{Y}_{j,i}^{op}$ holds a superior position compared to $\:{Y}_{j,i}$, it will be substituted.

#### Chaos map

The chaos map approach relies on the use of chaotic variables, which are characterized by their inherent unpredictability, as a replacement for random variables. Chaos sequences, which are bounded and non-periodic, are commonly observed in dynamic, nonlinear systems^30^. These sequences have a higher convergence rate compared to other probability-based random searches. By incorporating chaotic variables into the osprey optimizer, exploration within the solution space can be improved due to the dynamic nature of the turbulence sequence.

Various optimization methods utilize different chaotic maps that possess unique characteristics. Modifying the starting conditions of these maps can generate diverse sequences. In this study, the sinusoidal chaotic map is employed to enhance the convergence speed of the osprey optimizer. This approach strikes a balance between exploitation and exploration, leading to improved outcomes in the search for solutions within the solution space. Consequently, the issue of getting trapped in local optima is avoided.

To enhance the osprey optimizer process, a modification has been made by replacing the use of random numbers with random numbers generated by the Chaos function. The formulation of the sinusoidal map is provided below:

23 $$\:{Y}_{j,i}^{P1}={Y}_{j,i}+{r}_{j,i}\times\:\left(H{G}_{j,i}-(1+{\theta\:}_{j,i})\right)\times\:{Y}_{j,i}$$

where,

24 $$\:{\theta\:}_{j,i+1}=x.{\theta\:}_{j,i}^{2}\text{sin}\left(\pi\:.{\theta\:}_{j,i}\right)$$

where, $\:{\theta\:}_{j,i+1}$ specifies a chaotic value in the current iteration, and $\:x=2.3$ is a constant value, defining the switch parameter. The initial value of $\:{\theta\:}_{j,0}$ is assumed to be 0.5.

### Algorithm validation

To validate the effectiveness of the Promoted Osprey Optimizer (POO), comprehensive benchmarking tests were conducted using the “CEC-BC-2019 test suite” from “The 100-Digit Challenge.” The validation process involved comparing the performance of the POO against five established metaheuristic algorithms across a series of function optimization tasks. Each function from the suite represents a different optimization challenge, ranging from simple to highly complex landscapes, and is designated as either unimodal, multimodal, hybrid, or composition according to its characteristics.

The functions are as follows:

- Unimodal Functions (Simple Landscapes):

CEC01: 2-dimensional function, ideal for testing the exploitation capabilities of the algorithms.

CEC02: 3-dimensional function, slightly more complex, further challenging the exploitation phases.

- Multimodal Functions (Complex Landscapes with Multiple Optima):

CEC03: 5-dimensional function, used to assess the exploration strategies due to its numerous local optima.

CEC04: 100-dimensional function that provides a rigorous test of the optimizer’s capability to discriminate between various high-quality solutions and find the global optimum.

- Hybrid Functions (Combinations of Unimodal or Multimodal):

CEC05 and CEC06: Both are 100-dimensional problems that evaluate the algorithm’s ability to adapt to varied landscapes within a single optimization task.

- Composition Functions (Integrated Complex Landscapes):

CEC07 to CEC10: These are 100-dimensional functions representing some of the most challenging scenarios with a mixture of landscapes integrated through weighted sums.

Validation Process.

The validation process was carried out by executing each algorithm on the aforementioned test functions, ensuring that the algorithms were run under the same computational conditions. Several trials were conducted for each scenario to account for the stochastic nature of the algorithms.

The POO was benchmarked against five metaheuristic algorithms, including Teamwork Optimization Algorithm (TOA)^31^, Harris Hawks Optimization (HHO)^32^, Supply-Demand-Based Optimization (SDO)^33^, White Shark Optimizer (WSO)^34^, and the traditional Osprey Optimizer (OO). The algorithm parameter values of the compared techniques are presented in Table 1.

**Table 1:** Algorithm parameter value of the studied techniques.

Algorithm	Parameter	Value
Teamwork Optimization Algorithm (TOA) [31]	$\:t$	0.2×N
	$\:l$	0.5
	$\:f$	0.5
	$\:c$	0.8
	$\:e$	0.2
Harris Hawks Optimization (HHO) [32]	$\:{E}_{0}$	$\:0.5$
	$\:{E}_{1}$	$\:1$
Supply-Demand-Based Optimization (SDO) [33]	$\:{a}_{1}$	$\:1.5$
White Shark Optimizer (WSO) [34]	$\:f$	[0.07, 0.75]
	$\:\tau\:$	4.11
	$$\:p$$	[0.5, 1.5]
	$\:{a}_{0}$	6.25
	$\:{a}_{1}$	100
	$\:{a}_{2}$	0.0005
Osprey Optimizer (OO)	$\:P$	0.5

The selected values of the control parameters were determined through an extensive review of existing literature on prior studies that utilized these algorithms for comparable optimization challenges. Additionally, empirical testing has shown that these values yield effective performance for the Optimal Reactive Power Dispatch (ORPD) problem, thereby establishing them as an appropriate selection for this particular application. In this research, specific population sizes for all algorithms is set to 50 individuals. All the algorithms were evaluated 15 times for providing a fair analysis.

The performance metrics collected were as follows:

MF: The mean fitness value.

SD: The standard deviation value.

Table 2 presents a comparative analysis of the recommended osprey optimizer in comparison with the other evaluated algorithms.

**Table 2:** Comparative analysis of the recommended osprey optimizer in comparison with the other evaluated algorithms.

	POO		OO		TOA	
	MF	SD	MF	SD	MF	SD
CEC01	4155.099	17106.23	5001.213	19703.39	1.6E + 10	9.99E + 09
CEC02	3.453373	0	3.553918	0	14.77173	0
CEC03	9.658773	0	10.43621	0	10.6678	0
CEC04	25.83189	14.17874	33.40389	17.47579	77.41824	95.81583
CEC05	0.069748	0.073767	0.075811	0.086848	1.098129	0.172105
CEC06	0.353153	1.516641	0.363624	1.84887	3.838593	1.68177
CEC07	10.4008	90.5373	13.13092	112.539	404.4611	206.7346
CEC08	0.244836	4.577539	0.263939	4.992015	4.273898	0.39597
CEC09	1.409065	0	1.796463	0	2.562367	0.303659
CEC10	2.268995	0	2.364082	0	16.26713	0.116775
	HHO		SDO		WSO	
	MF	SD	MF	SD	MF	SD
CEC01	27,933,188	5,888,665	4343.774	19018.34	4.56E + 10	6.26E + 10
CEC02	14.81719	0	3.272729	0	64.32058	72.8151
CEC03	9.889406	0	12.24889	0	11.81964	0.000828
CEC04	74.46162	88.64707	28.86324	33.34913	289.5465	365.931
CEC05	1.131585	0.148169	1.666987	0.06671	1.9353	0.300371
CEC06	3.731789	1.616768	9.19396	0.571005	8.543823	1.30963
CEC07	402.8202	214.842	106.317	12.27217	516.2686	289.1294
CEC08	4.831865	0.337616	5.35531	0.605295	5.641925	0.398882
CEC09	2.689816	0.308875	1.491277	0	4.592122	2.686101
CEC10	17.83356	0.115708	2.565199	0	16.32242	0.154871

Analyzing the results, it can be observed that the POO consistently outperforms the other algorithms in terms of both MF and SD in most of the optimization tasks. This indicates that the POO is more effective in finding optimal solutions and exhibits more stability in its performance. For example, in CEC01, the POO achieves a significantly lower MF value compared to all other algorithms, indicating better optimization. Similarly, in CEC02 and CEC03, the POO demonstrates superior performance by achieving lower MF values and zero SD values, implying greater consistency in its results. Furthermore, in CEC04, the POO performs better than the traditional OO and TOA in terms of both MF and SD, suggesting its ability to handle complex optimization challenges.

In CEC07, the POO outperforms all other algorithms in terms of MF, demonstrating its effectiveness in solving multimodal optimization problems. Overall, the results of Table 2 indicate the superiority of the proposed POO algorithm over the other evaluated algorithms. Its consistent performance across various optimization tasks, lower MF values, and smaller SD values highlight its effectiveness and stability. These findings suggest that the POO has the potential to be a reliable and efficient metaheuristic algorithm for solving a wide range of optimization problems.

## Results and discussions

The study utilized MATLAB R2019b as the programming platform and relied on the Matpower toolbox for importing the test system data. The simulations were conducted on a computer with an Intel i7-6700HQ Asus N552 processor and 12GB RAM, operating in the Matlab 2018b environment. The pseudocode of this study is given in Algorithm 1.

**Table:** 

Algorithm 1: Promoted Osprey Optimizer (POO)
*Input*: *- System data (bus data*,* line data*,* generator data*,* etc.)* *- Number of ospreys (N)* *- Maximum number of iterations (Max_Iter)* *- Convergence tolerance (Tol)*
*Output*: *- Optimal reactive power dispatch (ORPD) solution* *Initialization*: *- Initialize osprey positions (x) and velocities (v) randomly* *- Initialize the best position (x_best) and the best fitness (f_best)* *Main Loop*: *- For each iteration (i = 1 to Max_Iter)* *- Evaluate the fitness (f) of each osprey using the objective function* *- Update the best position (x_best) and the best fitness (f_best) if a better solution is found* *- Update the osprey positions (x) and velocities (v) using the following equations*: x_new = x_old + v_old + r1 * (x_best - x_old) + r2 * (x_global_best - x_old) v_new = w * v_old + r1 * (x_best - x_old) + r2 * (x_global_best - x_old) *- Apply boundary constraints to the osprey positions (x)* *Termination*: *- If the convergence tolerance (Tol) is met or the maximum number of iterations (Max_Iter) is reached*,* terminate the algorithm* *Output*: *- Return the optimal reactive power dispatch (ORPD) solution*

In the following, the results of the method have been analyzed and discussed.

### Minimizing the loss value

This section discusses the results of simulations that were conducted to assess the effectiveness of a method for Optimal Reactive Power Dispatch (ORPD) applied to two different electrical power systems as defined by the Institute of Electrical and Electronics Engineers (IEEE), one with 57 buses and another with 118 buses. A “bus” is a node where one or more lines start or end in a power system network, not a vehicle. The performance of the ORPD method was compared with four other recent advanced techniques in this field:

- Fractional Calculus with Particle Swarm Optimization Gravitational Search Algorithm (FPSOGSA)^19^.
- Sine-Cosine Algorithm (SCA) algorithm^20^.
- Canonical Differential Evolutionary Particle Swarm Optimizer (C-DEEPSO)^21^.
- Enhanced Transient Search Optimization (ETSO)^23^.

These optimization strategies are employed to discover the most optimal arrangement of variables in a reactive power dispatch scenario. Reactive power dispatch plays a vital role in the management and operation of an electric power system, with a focus on minimizing power losses and enhancing voltage profiles while adhering to various system constraints. The test systems, which adhere to IEEE standards, offer standardized datasets that enable researchers to evaluate diverse optimization techniques and assess the efficiency of their proposed solutions under comparable circumstances.

#### IEEE 57-bus

The IEEE 57-bus system serves as a conventional benchmark for conducting power flow analysis and optimization. Comprising 57 buses, 7 generators, 3 transformers, 42 loads, and 80 transmission lines, it provides a comprehensive framework for evaluating and enhancing power system performance.

The system comprises reactive and active power components, specifically 233.8 MVAR and 1250.4 MW, respectively. The total generation capacity slightly exceeds the demand, with 1275.4 MW of active power and 263.6 MVAR of reactive power. Operating at a voltage of 138 kV, the Optimal Reactive Power Dispatch (ORPD) aims to achieve two primary objectives in this scenario.

Firstly, it aims to reduce or eliminate active power losses (case 1), which plays a significant role in enhancing the efficiency of the power system. Secondly, it focuses on minimizing voltage deviations (fluctuations) at the load buses (case 2), as this is crucial for maintaining power supply stability and preserving the integrity of connected equipment.

In order to achieve the stated objectives and ensure that the system operates according to specific standards, penalties are incorporated into the objective function in the event of any violations of constraints. The control of the system is determined by a set of 27 control parameters, which include the following:

- VAR (volt-ampere reactive) adjustments, which must fall within the range of -20 to + 20 MVAR. These adjustments allow for precise fine-tuning of the reactive power in the system.
- Voltage levels at specific buses (nodes) within the power grid network, namely buses number 1, 2, 3, 6, 8, and 9.
- Tap settings of transformers, which must be adjusted within a specific range of 0.9 to 1.1. This adjustment impacts the voltage levels and power flow through the transformers.
- Reactive power compensators located at buses 12-, 18-, and 25-, as well as bus 53-. These compensators assist in managing the reactive power within the system.
- Voltage magnitudes throughout the entire system must remain within a predefined range of 0.94 to 1.06 per unit (p.u.), where 1 p.u. typically represents the system’s rated voltage. All fifteen transformers have tap settings that must be correctly set within the given parameters.

This meticulous control of various parameters is crucial for optimizing the system’s performance, minimizing inefficiencies, and maintaining voltage stability.

**Fig. 1:**
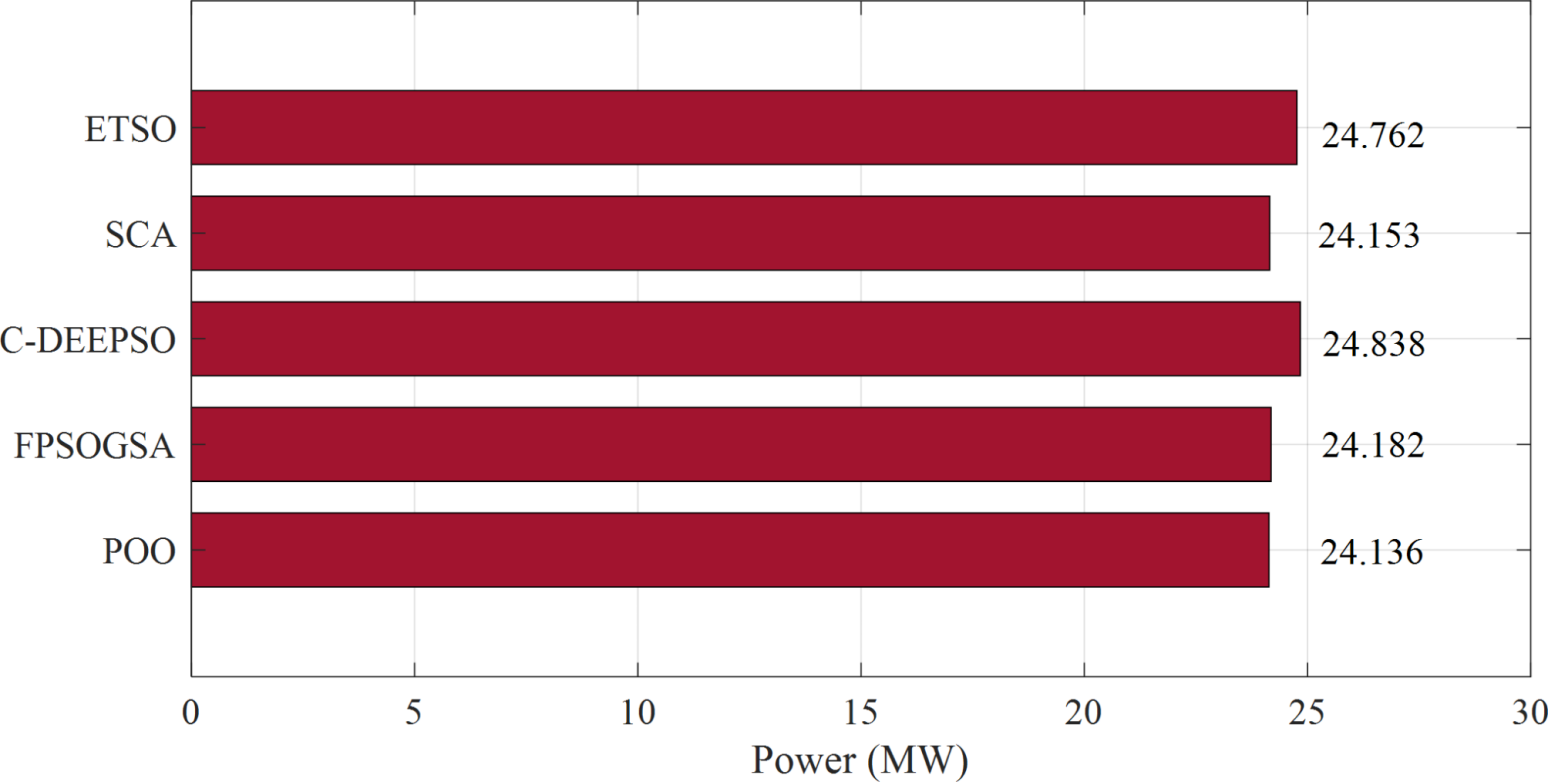
Minimizing power loss in the 57-bus system through a comparison between the proposed POO algorithm and various optimization techniques.

Figure 1 displays the objective values of minimizing power loss in the 57-bus system through a comparison between the proposed Promoted Osprey Optimizer (POO) and various optimization techniques, include Fractional Calculus with Particle Swarm Optimization Gravitational Search Algorithm (FPSOGSA), Sine-Cosine Algorithm (SCA), Canonical Differential Evolutionary Particle Swarm Optimizer (C-DEEPSO), and Enhanced Transient Search Optimization (ETSO).

**Fig. 2:**
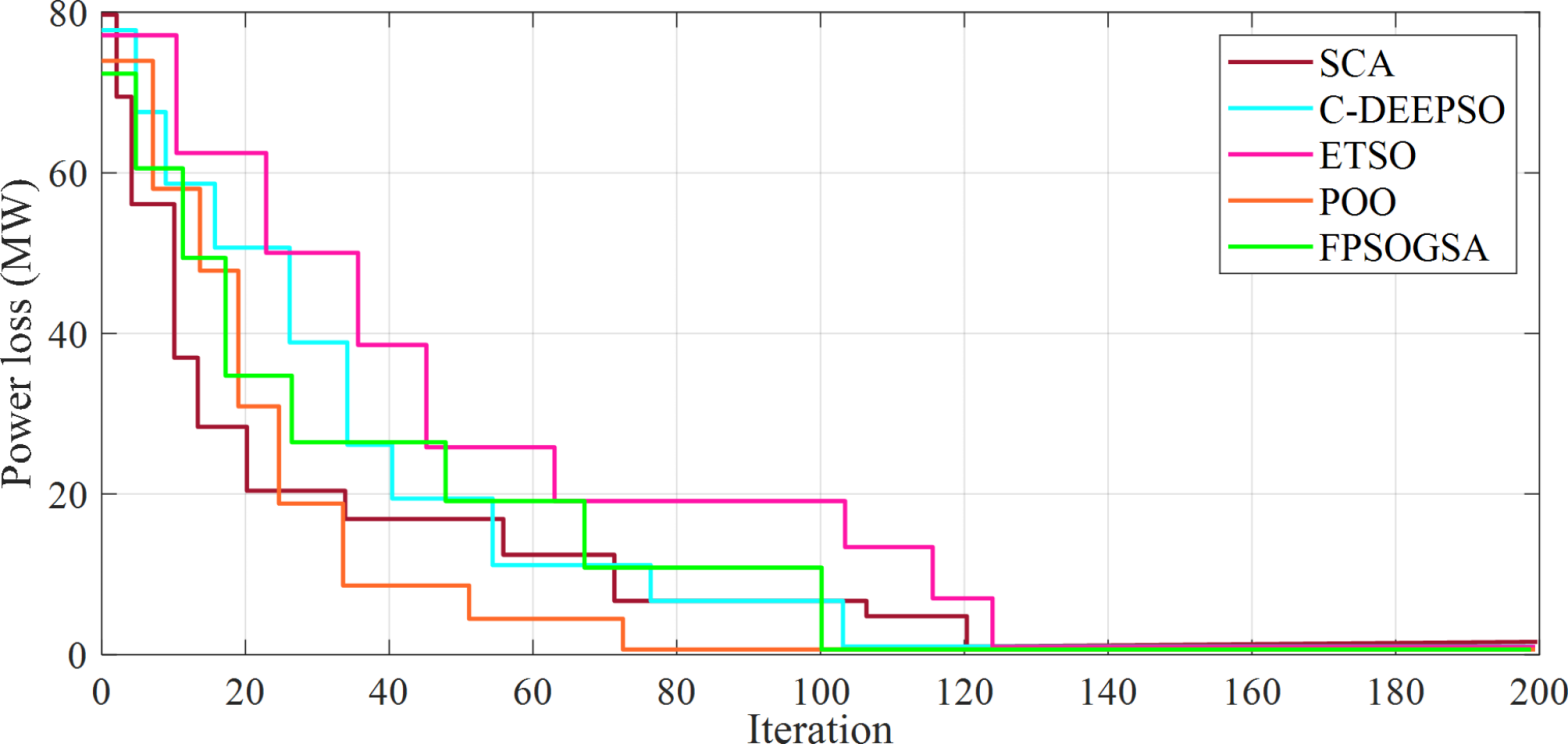
Convergence analysis of the power loss for the 57-bus system.

The findings presented in Fig. 1 demonstrated that the proposed POO algorithm outperformed other optimization techniques in terms of power loss reduction in the 57-bus system. Compared to FPSOGSA, the POO algorithm achieved a power loss reduction of 0.046 MW. Similarly, it reduced power loss by 0.702 MW compared to C-DEEPSO, 0.017 MW compared to SCA, and 0.626 MW compared to ETSO. These results indicated that the POO algorithm effectively optimized reactive power dispatch and enhanced power system performance with EV integration. Additionally, the POO algorithm exhibited faster and more stable convergence, as depicted in Fig. 2. Overall, these results highlighted the superior and robust nature of the POO algorithm in solving the ORPD problem with EVs.

Table 3 presented an extensive examination of the optimal design parameters for the 57-bus system. This analysis encompassed voltage levels, VAR compensators, transformer tap settings, and VAR adjustments.

**Table 3:** Extensive examination of the optimal design parameters for the 57-bus system.

Design Variables	Value	Design Variables	Value
$\:{\left|V\right|}_{\text{b}\text{u}\text{s}\#1}$	1.065	$\:Transforme{r}_{branch\#36}$	0.965
$\:{\left|V\right|}_{\text{b}\text{u}\text{s}\#2}$	1.003	$\:Transforme{r}_{branch\#37}$	1.024
$\:{\left|V\right|}_{\text{b}\text{u}\text{s}\#3}$	0.956	$\:Transforme{r}_{branch\#41}$	0.969
$\:{\left|V\right|}_{\text{b}\text{u}\text{s}\#6}$	0.986	$\:Transforme{r}_{branch\#46}$	0.932
$\:{\left|V\right|}_{\text{b}\text{u}\text{s}\#8}$	0.971	$\:Transforme{r}_{branch\#54}$	0.959
$\:{\left|V\right|}_{\text{b}\text{u}\text{s}\#9}$	0.954	$\:Transforme{r}_{branch\#58}$	0.979
$\:{\left|V\right|}_{\text{b}\text{u}\text{s}\#12}$	0.969	$\:Transforme{r}_{branch\#59}$	0.936
$\:\:{Reactive\:power}_{\text{b}\text{u}\text{s}\#18}$	-1.886	$\:Transforme{r}_{branch\#65}$	0.935
$\:{Reactive\:power}_{\text{b}\text{u}\text{s}\#25}$	8.623	$\:Transforme{r}_{branch\#66}$	0.932
$\:{Reactive\:power}_{\text{b}\text{u}\text{s}\#53}$	19.787	$\:Transforme{r}_{branch\#71}$	0.934
$\:Transforme{r}_{branch\#19}$	0.905	$\:Transforme{r}_{branch\#73}$	0.999
$\:Transforme{r}_{branch\#20}$	1.004	$\:Transforme{r}_{branch\#76}$	0.987
$\:Transforme{r}_{branch\#31}$	1.011	$\:Transforme{r}_{branch\#80}$	0.943
$\:Transforme{r}_{branch\#35}$	1.057		

A minus value for reactive power value at a bus, indicating that it was absorbing reactive power from the system, could have several negative consequences, including voltage instability, increased power losses, and reduced system reliability, as the reactive power absorption of bus could disrupt the balance of the system and lead to a range of issues that could compromise its overall performance and stability.

The achieved values of these parameters were established by solving an ORPD problem. This problem utilized diverse optimization techniques to identify the most appropriate values that fulfilled the desired objectives and operational constraints. Figure 2 illustrated the convergence analysis of the power loss for the 57-bus system during 200 iterations.

Each iteration represented a step towards finding the optimal solution. The x-axis denoted the number of iterations, while the y-axis illustrated the power loss value. As can be observed, the objective function value experienced a slower decrease as the algorithm approached convergence, indicating the achievement of the optimal solution.

#### IEEE 118-bus system

The IEEE 118-bus system serves as a standardized test system for power flow analysis and optimization purposes. It comprises 57 buses, 7 generators, 3 transformers, 42 loads, and 80 transmission lines. The system’s data and diagram can be accessed through the provided link. This system is a simplified representation of the American Electric Power system located in the U.S. Midwest, specifically it existed in December 1962. With numerous voltage control devices and a robust nature, the system demonstrated convergence within approximately 5 iterations using a fast decoupled power flow method. Due to its reliability and versatility, the system had extensive application in testing novel algorithms, technologies, and control schemes for power systems operation and planning.

The system had a combined generating capacity of 263.6 MVAR and 1275.4 MW and a total load demand of 233.8 MVAR and 1250.4 MW. The voltage magnitudes should be between 0.94 and 1.06 per unit (p.u.), and the transformer tap settings should be in the interval [0.9, 1.1]. The VAR compensations should be in the interval [-20, 20] MVARs. The operational voltage of the system was 138 kV^35^.

**Fig. 3:**
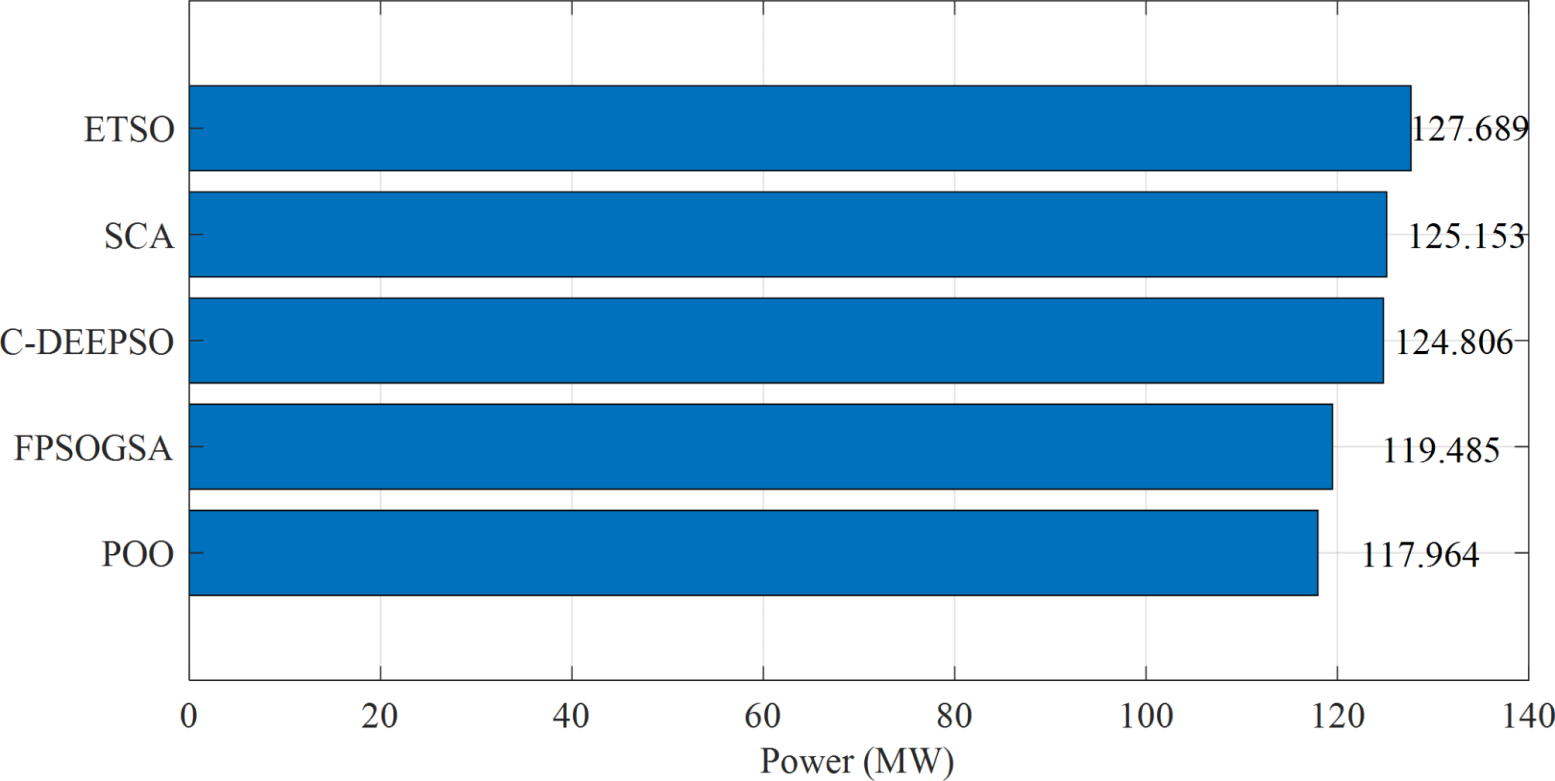
Minimizing power loss in the 118-bus system through a comparison between the proposed POO algorithm and various optimization techniques.

Figure 3 displayed the objective values of minimizing power loss in the IEEE 118-bus system through a comparison between the proposed Promoted Osprey Optimizer (POO) and various optimization techniques that were mentioned before.

**Fig. 4:**
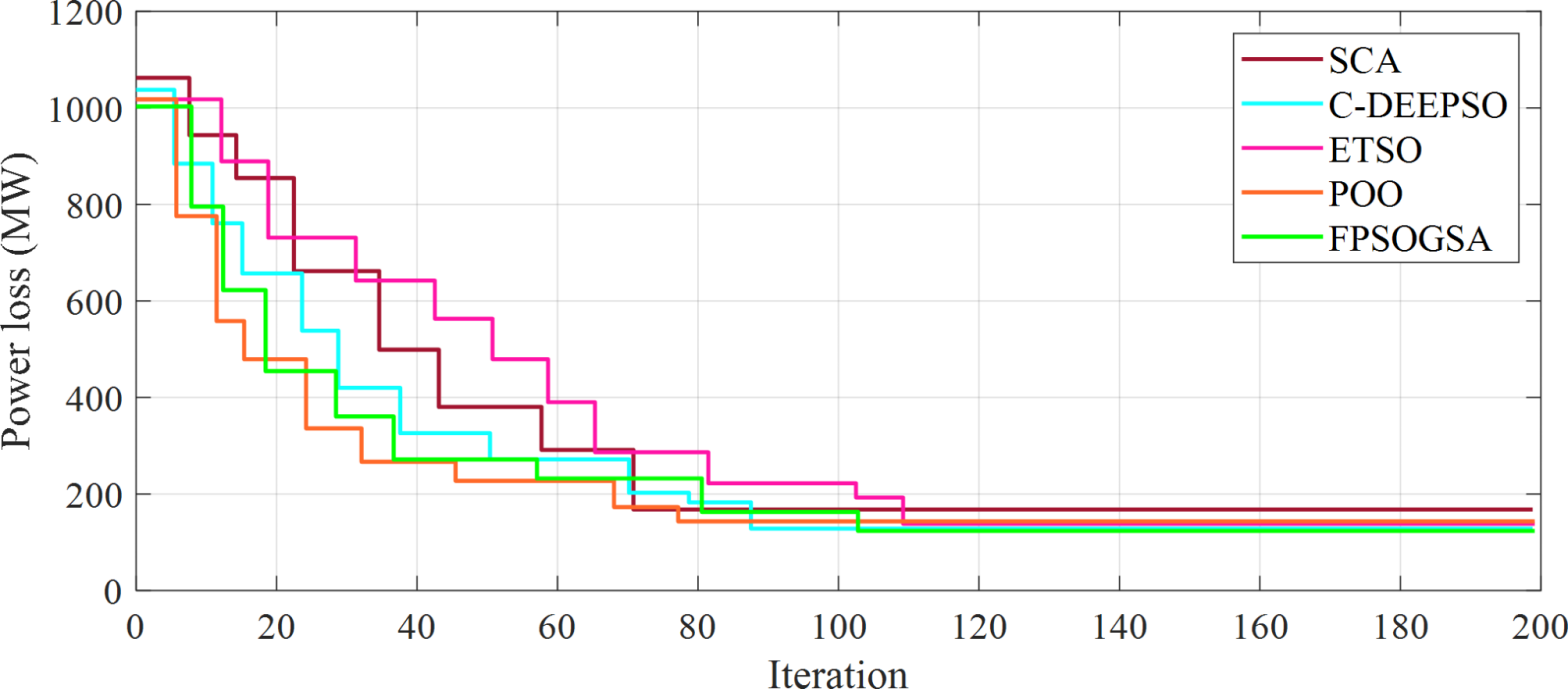
Curves of convergence of the power loss for the 118-bus system over a span of 200 iterations.

The findings from Fig. 3 demonstrate that the proposed POO algorithm outperformed other optimization techniques in terms of power loss reduction in the IEEE 118-bus system. Compared to FPSOGSA, the POO algorithm achieved a power loss reduction of 1.521 MW. Additionally, it reduced power loss by 6.842 MW compared to C-DEEPSO, 7.189 MW compared to SCA, and 9.725 MW compared to ETSO. These results highlighted the effectiveness of the POO algorithm in optimizing reactive power dispatch and enhancing power system performance with EV integration. Furthermore, the POO algorithm exhibited faster and more stable convergence than other techniques, as depicted in Fig. 4. Overall, these results demonstrated the superior and robust nature of the POO algorithm in addressing the ORPD problem with EVs.

Table 4 presents an extensive examination of the optimal design parameters for the 118-bus system. This analysis encompasses voltage levels, VAR compensators, transformer tap settings, and VAR adjustments.

**Table 4:** Extensive examination of the optimal design parameters for the 118-bus system.

Design variables	Value	Design variables	Value
$\:{\left|V\right|}_{\text{b}\text{u}\text{s}\#1}$	0.950	|V| at bus 89	0.931
$\:{\left|V\right|}_{\text{b}\text{u}\text{s}\#4}$	0.943	|V| at bus 90	0.948
$\:{\left|V\right|}_{\text{b}\text{u}\text{s}\#6}$	1.021	|V| at bus 91	0.945
$\:{\left|V\right|}_{\text{b}\text{u}\text{s}\#8}$	0.932	|V| at bus 92	1.073
$\:{\left|V\right|}_{\text{b}\text{u}\text{s}\#10}$	0.982	|V| at bus 99	1.034
$\:{\left|V\right|}_{\text{b}\text{u}\text{s}\#12}$	1.050	|V| at bus 100	0.952
$\:{\left|V\right|}_{\text{b}\text{u}\text{s}\#15}$	0.998	|V| at bus 103	1.001
$\:{\left|V\right|}_{\text{b}\text{u}\text{s}\#18}$	0.922	$\:{\left|V\right|}_{\text{b}\text{u}\text{s}\#104}$	0.951
$\:{\left|V\right|}_{\text{b}\text{u}\text{s}\#19}$	1.051	$\:{\left|V\right|}_{\text{b}\text{u}\text{s}\#105}$	0.956
$\:{\left|V\right|}_{\text{b}\text{u}\text{s}\#24}$	0.940	$\:{\left|V\right|}_{\text{b}\text{u}\text{s}\#107}$	0.956
$\:{\left|V\right|}_{\text{b}\text{u}\text{s}\#25}$	0.959	$\:{\left|V\right|}_{\text{b}\text{u}\text{s}\#110}$	0.931
$\:{\left|V\right|}_{\text{b}\text{u}\text{s}\#26}$	0.944	$\:{\left|V\right|}_{\text{b}\text{u}\text{s}\#111}$	1.086
$\:{\left|V\right|}_{\text{b}\text{u}\text{s}\#27}$	1.065	$\:{\left|V\right|}_{\text{b}\text{u}\text{s}\#112}$	0.952
$\:{\left|V\right|}_{\text{b}\text{u}\text{s}\#31}$	0.944	$\:{\left|V\right|}_{\text{b}\text{u}\text{s}\#113}$	0.927
$\:{\left|V\right|}_{\text{b}\text{u}\text{s}\#32}$	1.068	$\:{\left|V\right|}_{\text{b}\text{u}\text{s}\#116}$	0.957
$\:{\left|V\right|}_{\text{b}\text{u}\text{s}\#34}$	0.959	$\:VA{R}_{\text{b}\text{u}\text{s}\#5}$	6.588
$\:{\left|V\right|}_{\text{b}\text{u}\text{s}\#36}$	0.933	$\:VA{R}_{\text{b}\text{u}\text{s}\#34}$	1.538
$\:{\left|V\right|}_{\text{b}\text{u}\text{s}\#40}$	0.938	$\:VA{R}_{\text{b}\text{u}\text{s}\#37}$	14.225
$\:{\left|V\right|}_{\text{b}\text{u}\text{s}\#42}$	0.967	$\:VA{R}_{\text{b}\text{u}\text{s}\#44}$	5.204
$\:{\left|V\right|}_{\text{b}\text{u}\text{s}\#46}$	0.925	$\:VA{R}_{\text{b}\text{u}\text{s}\#45}$	0.551
$\:{\left|V\right|}_{\text{b}\text{u}\text{s}\#49}$	1.057	$\:VA{R}_{\text{b}\text{u}\text{s}\#46}$	3.238
$\:{\left|V\right|}_{\text{b}\text{u}\text{s}\#54}$	1.064	$\:VA{R}_{\text{b}\text{u}\text{s}\#48}$	0.801
$\:{\left|V\right|}_{\text{b}\text{u}\text{s}\#55}$	1.067	$\:VA{R}_{\text{b}\text{u}\text{s}\#74}$	0.177
$\:{\left|V\right|}_{\text{b}\text{u}\text{s}\#56}$	0.987	$\:VA{R}_{\text{b}\text{u}\text{s}\#79}$	2.054
$\:{\left|V\right|}_{\text{b}\text{u}\text{s}\#59}$	0.925	$\:VA{R}_{\text{b}\text{u}\text{s}\#82}$	0.837
$\:{\left|V\right|}_{\text{b}\text{u}\text{s}\#61}$	0.948	$\:VA{R}_{\text{b}\text{u}\text{s}\#83}$	0.898
$\:{\left|V\right|}_{\text{b}\text{u}\text{s}\#62}$	1.006	$\:VA{R}_{\text{b}\text{u}\text{s}\#105}$	1.002
$\:{\left|V\right|}_{\text{b}\text{u}\text{s}\#65}$	0.935	$\:VA{R}_{\text{b}\text{u}\text{s}\#107}$	1.018
$\:{\left|V\right|}_{\text{b}\text{u}\text{s}\#66}$	1.025	$\:VA{R}_{\text{b}\text{u}\text{s}\#110}$	1.114
$\:{\left|V\right|}_{\text{b}\text{u}\text{s}\#69}$	0.962	$\:Transformer\:se{t}_{line\#8}$	0.897
$\:{\left|V\right|}_{\text{b}\text{u}\text{s}\#70}$	1.013	$\:Transformer\:se{t}_{line\#32}$	0.906
$\:{\left|V\right|}_{\text{b}\text{u}\text{s}\#72}$	1.081	$\:Transformer\:se{t}_{line\#36}$	1.093
$\:{\left|V\right|}_{\text{b}\text{u}\text{s}\#73}$	0.949	$\:Transformer\:se{t}_{line\#51}$	0.917
$\:{\left|V\right|}_{\text{b}\text{u}\text{s}\#74}$	1.027	$\:Transformer\:se{t}_{line\#93}$	1.118
$\:{\left|V\right|}_{\text{b}\text{u}\text{s}\#76}$	0.922	$\:Transformer\:se{t}_{line\#95}$	0.924
$\:{\left|V\right|}_{\text{b}\text{u}\text{s}\#77}$	0.996	$\:Transformer\:se{t}_{line\#102}$	0.884
$\:{\left|V\right|}_{\text{b}\text{u}\text{s}\#80}$	0.933	$\:Transformer\:se{t}_{line\#107}$	0.913
$\:{\left|V\right|}_{\text{b}\text{u}\text{s}\#85}$	1.065	$\:Transformer\:se{t}_{line\#127}$	0.901
$\:{\left|V\right|}_{\text{b}\text{u}\text{s}\#87}$	0.936		

Figure 4 illustrates the convergence analysis of the power loss for the 118-bus system during 200 iterations.

Each iteration represented a step towards finding the optimal solution. As can be concluded, the objective function value experienced a slower decrease as the algorithm approached convergence, indicating the achievement of the optimal solution.

It should be noted that All algorithms reached convergence around the 100th iteration in both systems, even though the 118-bus system was approximately twice bigger than the 57-bus system in size. This convergence pattern could be explained by various factors, including the nature of the problem, which did not directly correlate with system size but was more closely associated with the number of variables and constraints. Of course, these were comparable in both systems. Moreover, the algorithms employed in this analysis were all population-based optimization techniques, specifically designed to effectively navigate the search space for the optimal solution, and their success in addressing complex optimization challenges was not inherently linked to the size of the system. Moreover, the parameters for each algorithm were meticulously adjusted to ensure optimal performance across both systems, involving modifications of aspects, such as population size, mutation rate, and crossover rate, among others. To provide a better analysis, the calculation times are listed in Table 5.

**Table 5:** The calculation times.

Algorithm	57-bus system (s)	118-bus system (s)
POO	23.4	51.2
FPSOGSA	34.6	73.1
SCA	31.2	61.5
C-DEEPSO	42.5	92.3
ETSO	51.8	114.9

The POO algorithm demonstrated the quickest calculation time across both systems, with the SCA algorithm being closely behind. In contrast, the C-DEEPSO and ETSO algorithms exhibited the longest calculation times, probably due to their more intricate search mechanisms.

### Minimizing the voltage deviation

#### IEEE 57-bus system

**Fig. 5:**
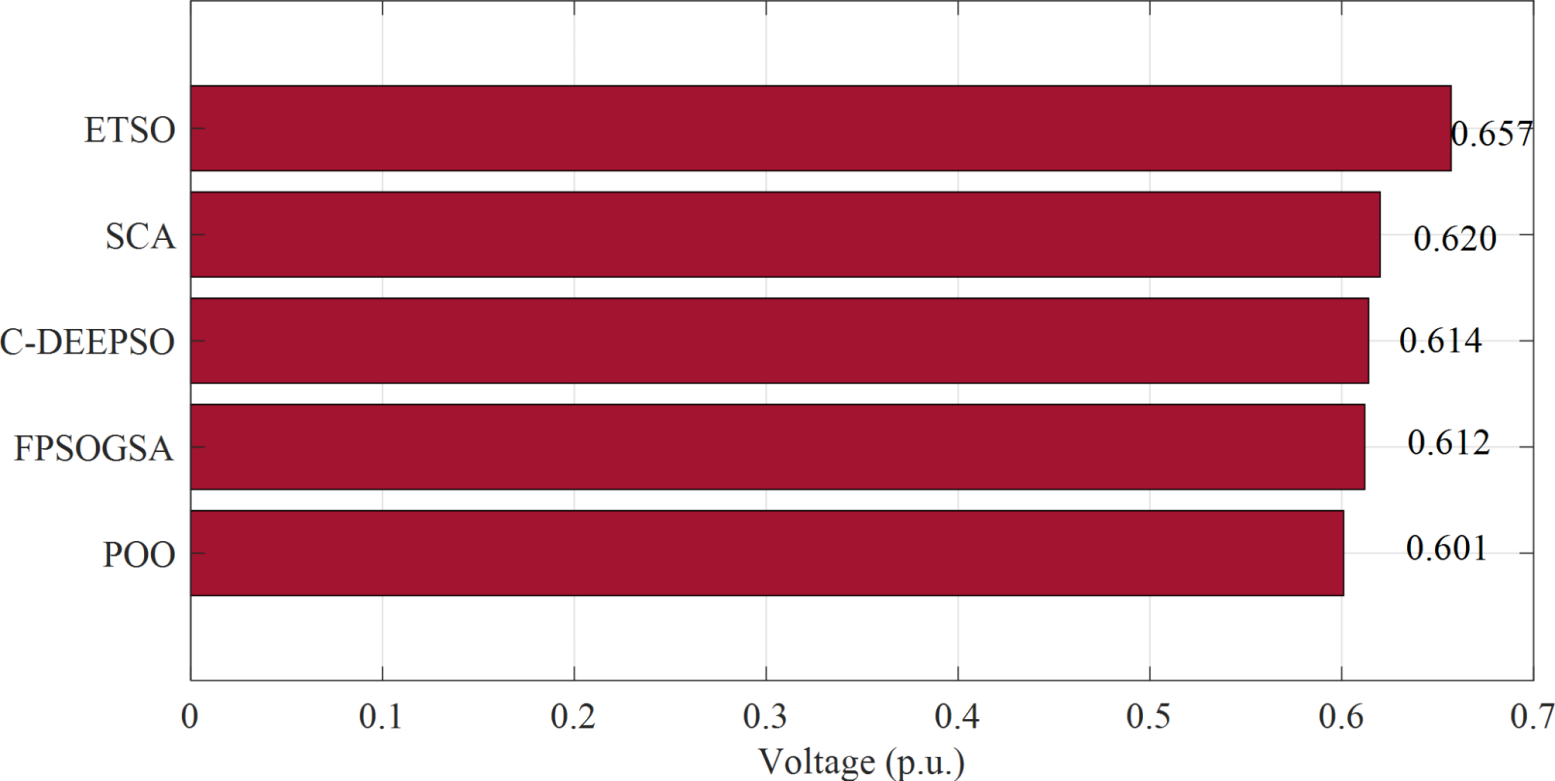
Minimizing Voltage deviation in the 57-bus system through a comparison between the proposed POO algorithm and various optimization techniques.

Figure 5 displays the objective values of minimizing voltage deviation in the 57-bus system through a comparison between the proposed Promoted Osprey Optimizer (POO) and various optimization techniques, such as Fractional Calculus with Particle Swarm Optimization Gravitational Search Algorithm (FPSOGSA), Sine-Cosine Algorithm (SCA), Canonical Differential Evolutionary Particle Swarm Optimizer (C-DEEPSO), and Enhanced Transient Search Optimization (ETSO).

The findings presented in Fig. 5 demonstrated that the proposed POO algorithm outperformed other optimization techniques in terms of minimizing voltage deviation in the 57-bus system. Voltage deviation refers to the extent to which bus voltages deviate from their nominal values, and it serves as an indicator of the power system’s voltage quality and stability. Compared to FPSOGSA, the POO algorithm reduced voltage deviation by 0.011 p.u., by 0.013 p.u. compared to C-DEEPSO, by 0.019 p.u. compared to SCA, and by 0.056 p.u. compared to ETSO.

**Fig. 6:**
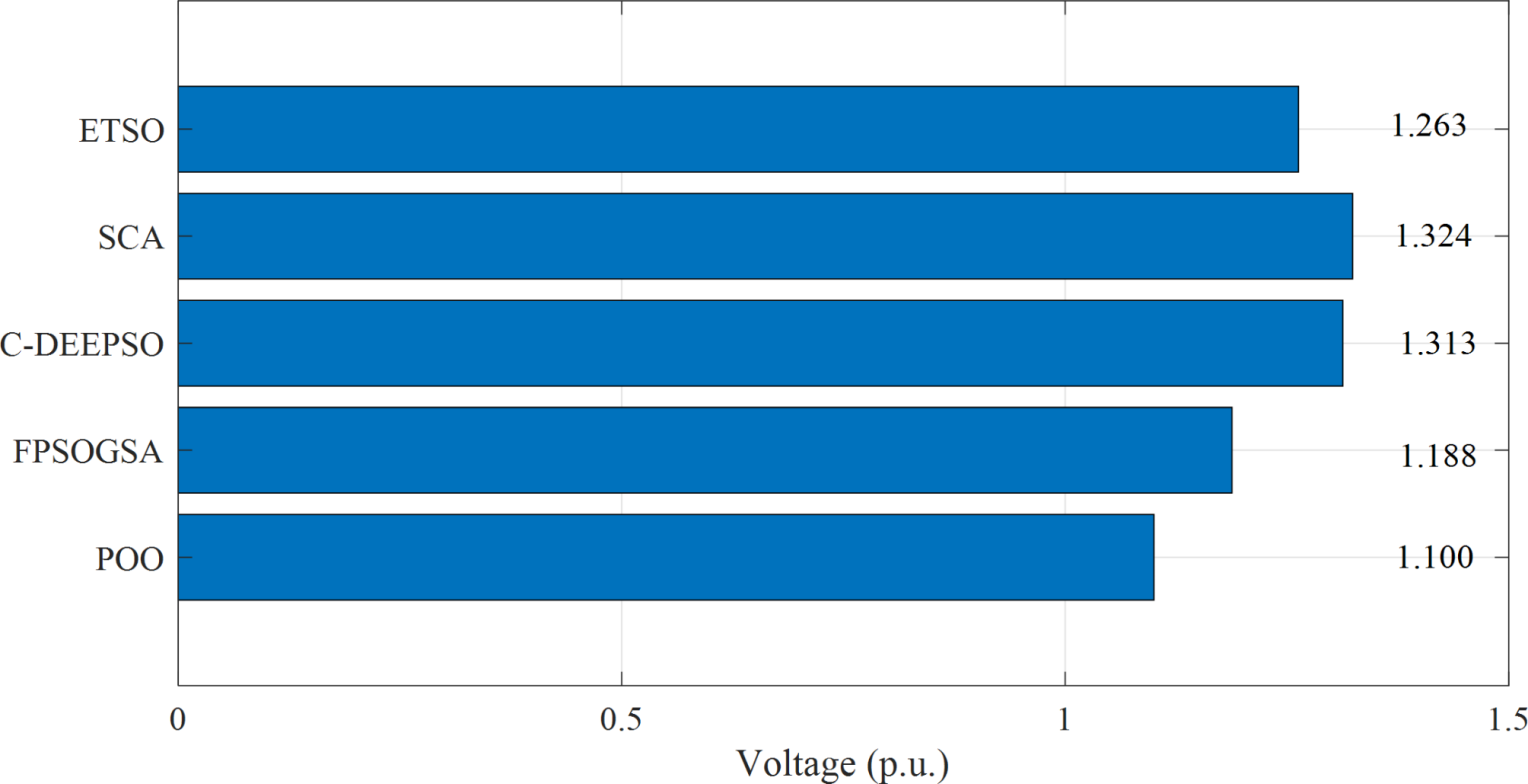
Minimizing Voltage deviation in the 118-bus system through a comparison between the proposed POO algorithm and various optimization techniques.

This highlighted the POO algorithm’s effectiveness in optimizing reactive power dispatch and enhancing the power system’s voltage profile and stability with EV integration. Additionally, the POO algorithm exhibited faster and more stable convergence than other techniques, as depicted in Fig. 6. These results underscored the POO algorithm’s superiority and robustness in addressing the ORPD problem with EVs. A thorough examination of the optimal design factors for reducing voltage deviation in the 57-bus system is given in Table 6.

**Table 6:** A thorough examination of the optimal design factors for reducing voltage deviation in the 57-bus system.

Design Variables	Value	Design Variables	Value
$\:{\left|V\right|}_{\text{b}\text{u}\text{s}\#1}$	1.025	$\:Transforme{r}_{branch\#36}$	0.909
$\:{\left|V\right|}_{\text{b}\text{u}\text{s}\#2}$	1.055	$\:Transforme{r}_{branch\#37}$	1.024
$\:{\left|V\right|}_{\text{b}\text{u}\text{s}\#3}$	1.009	$\:Transforme{r}_{branch\#41}$	0.950
$\:{\left|V\right|}_{\text{b}\text{u}\text{s}\#6}$	0.957	$\:Transforme{r}_{branch\#46}$	0.926
$\:{\left|V\right|}_{\text{b}\text{u}\text{s}\#8}$	0.953	$\:Transforme{r}_{branch\#54}$	0.917
$\:{\left|V\right|}_{\text{b}\text{u}\text{s}\#9}$	0.976	$\:Transforme{r}_{branch\#58}$	0.988
$\:{\left|V\right|}_{\text{b}\text{u}\text{s}\#12}$	1.021	$\:Transforme{r}_{branch\#59}$	0.993
$\:\:{Reactive\:power}_{\text{b}\text{u}\text{s}\#18}$	-1.431	$\:Transforme{r}_{branch\#65}$	0.999
$\:{Reactive\:power}_{\text{b}\text{u}\text{s}\#25}$	2.274	$\:Transforme{r}_{branch\#66}$	0.912
$\:{Reactive\:power}_{\text{b}\text{u}\text{s}\#53}$	16.916	$\:Transforme{r}_{branch\#71}$	0.950
$\:Transforme{r}_{branch\#19}$	1.002	$\:Transforme{r}_{branch\#73}$	1.039
$\:Transforme{r}_{branch\#20}$	0.989	$\:Transforme{r}_{branch\#76}$	0.905
$\:Transforme{r}_{branch\#31}$	0.982	$\:Transforme{r}_{branch\#80}$	0.970
$\:Transforme{r}_{branch\#35}$	1.089		

#### IEEE 118-bus system

Figure 6 illustrates the objective values of minimizing voltage deviation in the 118-bus system through a comparison between the proposed Promoted Osprey Optimizer (POO) and various optimization techniques, namely Fractional Calculus with Particle Swarm Optimization Gravitational Search Algorithm (FPSOGSA), Sine-Cosine Algorithm (SCA), Canonical Differential Evolutionary Particle Swarm Optimizer (C-DEEPSO), and Enhanced Transient Search Optimization (ETSO).

The findings presented in Fig. 6 demonstrated that the proposed POO algorithm outperformed other optimization techniques in achieving the lowest voltage in the 57-bus system. Voltage serves as a metric for measuring the electric potential difference between two points in the power system, and it reflects the overall power quality and efficiency of the system. In comparison with FPSOGSA, the POO algorithm reduced the voltage by 0.088 p.u. Furthermore, it achieved a reduction of 0.213 p.u. compared to C-DEEPSO, 0.224 p.u. compared to SCA, and 0.163 p.u. compared to ETSO.

These results indicated that the POO algorithm effectively optimized reactive power dispatch, leading to a decrease in power loss and voltage deviation in the power system with EV integration. Additionally, the POO algorithm exhibited faster and more stable convergence than other techniques, as depicted in Fig. 6. These outcomes highlighted the superior performance and robustness of the POO algorithm in addressing the ORPD problem with EVs.

Table 7 presents an extensive examination of the optimal design parameters for the 118-bus system.

**Table 7:** Extensive examination of the optimal design parameters for the 118-bus system.

Design variables	Value	Design variables	Value
$\:{\left|V\right|}_{\text{b}\text{u}\text{s}\#1}$	0.951	|V| at bus 89	0.945
$\:{\left|V\right|}_{\text{b}\text{u}\text{s}\#4}$	1.021	|V| at bus 90	1.030
$\:{\left|V\right|}_{\text{b}\text{u}\text{s}\#6}$	0.965	|V| at bus 91	1.021
$\:{\left|V\right|}_{\text{b}\text{u}\text{s}\#8}$	0.914	|V| at bus 92	1.003
$\:{\left|V\right|}_{\text{b}\text{u}\text{s}\#10}$	0.935	|V| at bus 99	0.987
$\:{\left|V\right|}_{\text{b}\text{u}\text{s}\#12}$	0.936	|V| at bus 100	0.995
$\:{\left|V\right|}_{\text{b}\text{u}\text{s}\#15}$	0.937	|V| at bus 103	0.958
$\:{\left|V\right|}_{\text{b}\text{u}\text{s}\#18}$	1.008	$\:{\left|V\right|}_{\text{b}\text{u}\text{s}\#104}$	0.928
$\:{\left|V\right|}_{\text{b}\text{u}\text{s}\#19}$	0.953	$\:{\left|V\right|}_{\text{b}\text{u}\text{s}\#105}$	0.969
$\:{\left|V\right|}_{\text{b}\text{u}\text{s}\#24}$	0.945	$\:{\left|V\right|}_{\text{b}\text{u}\text{s}\#107}$	0.976
$\:{\left|V\right|}_{\text{b}\text{u}\text{s}\#25}$	0.957	$\:{\left|V\right|}_{\text{b}\text{u}\text{s}\#110}$	1.043
$\:{\left|V\right|}_{\text{b}\text{u}\text{s}\#26}$	0.951	$\:{\left|V\right|}_{\text{b}\text{u}\text{s}\#111}$	0.928
$\:{\left|V\right|}_{\text{b}\text{u}\text{s}\#27}$	0.965	$\:{\left|V\right|}_{\text{b}\text{u}\text{s}\#112}$	0.953
$\:{\left|V\right|}_{\text{b}\text{u}\text{s}\#31}$	0.953	$\:{\left|V\right|}_{\text{b}\text{u}\text{s}\#113}$	0.954
$\:{\left|V\right|}_{\text{b}\text{u}\text{s}\#32}$	1.057	$\:{\left|V\right|}_{\text{b}\text{u}\text{s}\#116}$	1.018
$\:{\left|V\right|}_{\text{b}\text{u}\text{s}\#34}$	0.963	$\:VA{R}_{\text{b}\text{u}\text{s}\#5}$	0.000
$\:{\left|V\right|}_{\text{b}\text{u}\text{s}\#36}$	0.954	$\:VA{R}_{\text{b}\text{u}\text{s}\#34}$	0.485
$\:{\left|V\right|}_{\text{b}\text{u}\text{s}\#40}$	0.939	$\:VA{R}_{\text{b}\text{u}\text{s}\#37}$	1.861
$\:{\left|V\right|}_{\text{b}\text{u}\text{s}\#42}$	0.933	$\:VA{R}_{\text{b}\text{u}\text{s}\#44}$	0.581
$\:{\left|V\right|}_{\text{b}\text{u}\text{s}\#46}$	1.021	$\:VA{R}_{\text{b}\text{u}\text{s}\#45}$	1.558
$\:{\left|V\right|}_{\text{b}\text{u}\text{s}\#49}$	1.010	$\:VA{R}_{\text{b}\text{u}\text{s}\#46}$	0.825
$\:{\left|V\right|}_{\text{b}\text{u}\text{s}\#54}$	0.987	$\:VA{R}_{\text{b}\text{u}\text{s}\#48}$	1.325
$\:{\left|V\right|}_{\text{b}\text{u}\text{s}\#55}$	1.033	$\:VA{R}_{\text{b}\text{u}\text{s}\#74}$	0.501
$\:{\left|V\right|}_{\text{b}\text{u}\text{s}\#56}$	0.987	$\:VA{R}_{\text{b}\text{u}\text{s}\#79}$	0.974
$\:{\left|V\right|}_{\text{b}\text{u}\text{s}\#59}$	0.932	$\:VA{R}_{\text{b}\text{u}\text{s}\#82}$	1.070
$\:{\left|V\right|}_{\text{b}\text{u}\text{s}\#61}$	0.996	$\:VA{R}_{\text{b}\text{u}\text{s}\#83}$	1.024
$\:{\left|V\right|}_{\text{b}\text{u}\text{s}\#62}$	0.951	$\:VA{R}_{\text{b}\text{u}\text{s}\#105}$	0.958
$\:{\left|V\right|}_{\text{b}\text{u}\text{s}\#65}$	0.950	$\:VA{R}_{\text{b}\text{u}\text{s}\#107}$	0.981
$\:{\left|V\right|}_{\text{b}\text{u}\text{s}\#66}$	0.931	$\:VA{R}_{\text{b}\text{u}\text{s}\#110}$	1.029
$\:{\left|V\right|}_{\text{b}\text{u}\text{s}\#69}$	0.999	$\:Transformer\:se{t}_{line\#8}$	0.971
$\:{\left|V\right|}_{\text{b}\text{u}\text{s}\#70}$	0.944	$\:Transformer\:se{t}_{line\#32}$	0.914
$\:{\left|V\right|}_{\text{b}\text{u}\text{s}\#72}$	1.010	$\:Transformer\:se{t}_{line\#36}$	0.941
$\:{\left|V\right|}_{\text{b}\text{u}\text{s}\#73}$	1.028	$\:Transformer\:se{t}_{line\#51}$	0.911
$\:{\left|V\right|}_{\text{b}\text{u}\text{s}\#74}$	0.950	$\:Transformer\:se{t}_{line\#93}$	0.900
$\:{\left|V\right|}_{\text{b}\text{u}\text{s}\#76}$	0.959	$\:Transformer\:se{t}_{line\#95}$	0.996
$\:{\left|V\right|}_{\text{b}\text{u}\text{s}\#77}$	0.992	$\:Transformer\:se{t}_{line\#102}$	0.960
$\:{\left|V\right|}_{\text{b}\text{u}\text{s}\#80}$	1.021	$\:Transformer\:se{t}_{line\#107}$	0.975
$\:{\left|V\right|}_{\text{b}\text{u}\text{s}\#85}$	0.968	$\:Transformer\:se{t}_{line\#127}$	0.892
$\:{\left|V\right|}_{\text{b}\text{u}\text{s}\#87}$	0.940		

### Utilization of the POO algorithm for addressing the ORPD with EV charging stations

These charging stations are dynamic loads that vary based on the charging patterns of plug-in e-cars. To test the algorithm, the study uses the 57- and 118- bus systems, with 1000 plug-in e-cars added to buses 15 and 16 in each system.

Electric vehicles (EVs) equipped with plug-in technology are capable of traveling an estimated 25 miles, with an optimal operating temperature of 20 °C. Within this category of EVs, approximately 80% are sedan models.

**Fig. 7:**
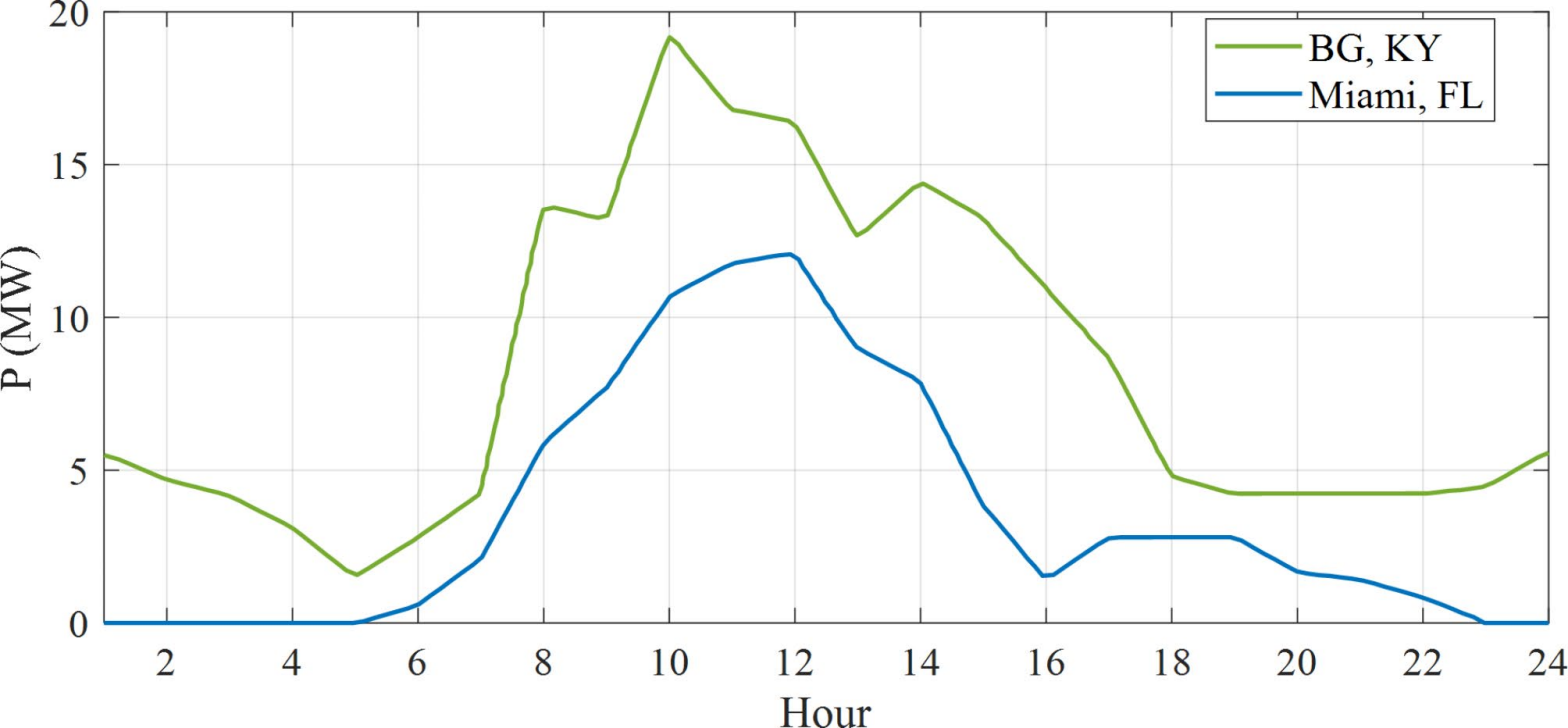
The fluctuations in power consumption for each plug on weekdays.

To capture the essence of their charging habits, data curves gracefully simulate the regional charging patterns of these plug-in e-cars in two distinguished locations, namely the enchanting Kentucky, Bowling Green, and the vibrant Miami, Florida, both are in the United States of America. The purpose was to enhance the ORPD problem by reducing voltage deviation cost and power loss, taking into account the influence of EV charging locations on the power system. The electric vehicle (EV) demand at 57-bus system trends in Bowling Green and Miami are illustrated in Fig. 7, highlighting the fluctuations in power consumption^36^.

**Fig. 8:**
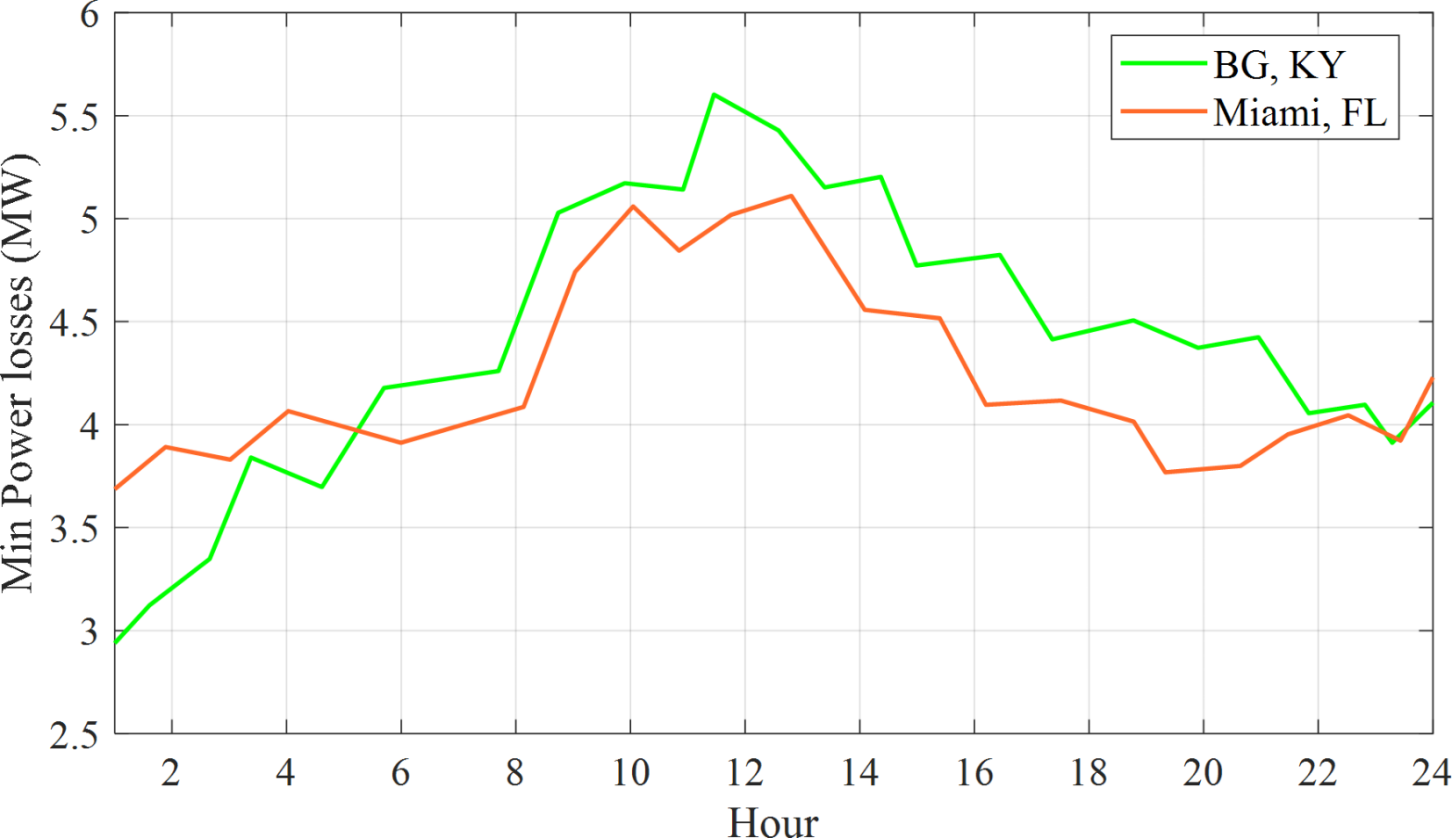
The 57-bus network encounters variations in power loss every hour.

The 57-bus network encounters variations in power loss every hour, as depicted in Fig. 8.

**Fig. 9:**
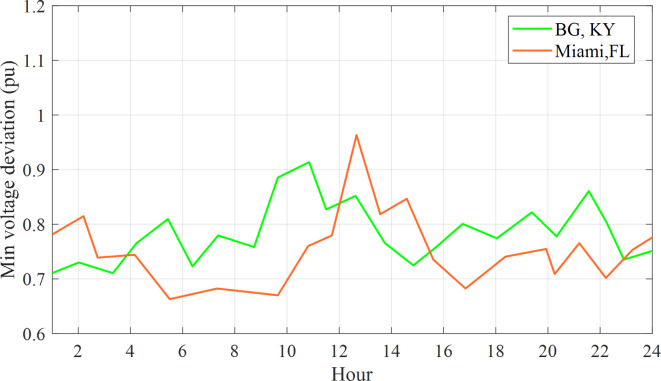
Hourly fluctuations in voltage deviation experienced by the 57-bus system.

Figure 8 demonstrates that the power loss reaches its minimum value at 19:00 AM in Miami, Florida, and at 19:00 PM in Bowling Green, Kentucky. Likewise, Fig. 9 illustrates the hourly variations in voltage deviation encountered by the 57-bus system.

**Fig. 10:**
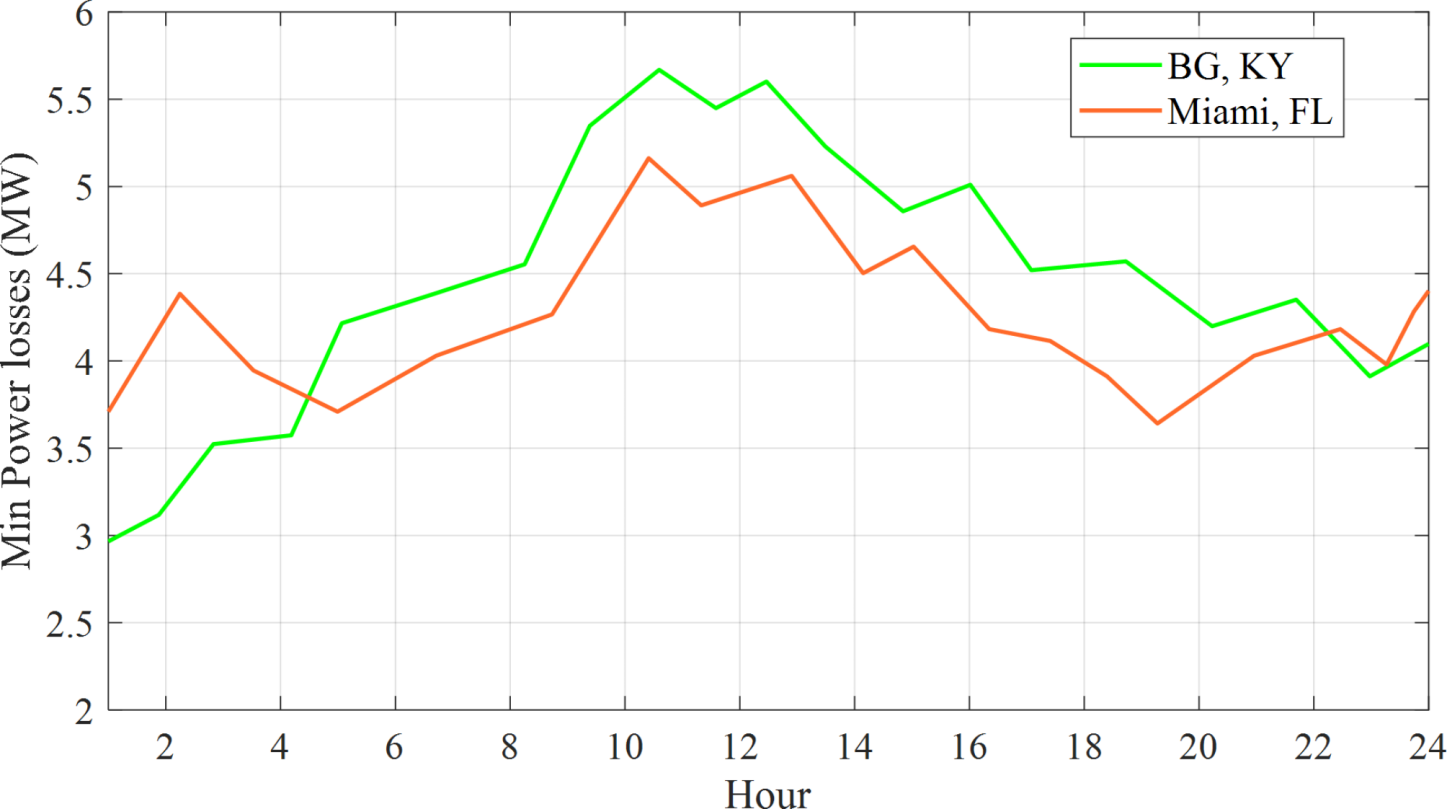
The variations of 118-bus network in power loss every hour.

Figure 9 shows that the voltage deviation reacheed its lowest point at 10:20 PM in Miami, Florida, and at 3:30 a.m. in Bowling Green, Kentucky. Additionally, the 118-bus network illustrated in Fig. 10 experienced hourly variations in power loss.

**Fig. 11:**
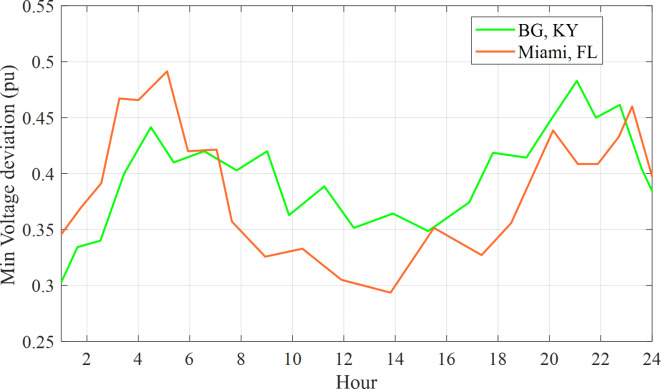
Hourly fluctuations in voltage deviation experienced by the 118-bus system.

As can be observed from Fig. 10, the minimum value of the power loss occurred at 19 p.m. in Miami, Florida, and at 1 a.m. in Bowling Green, Kentucky. Similarly, Fig. 11 shows the hourly fluctuations in voltage deviation experienced by the 118-bus system.

From Fig. 11, it is evident that the voltage deviation reached its lowest point at 1 a.m. in Bowling Green, Kentucky, and at 2 p.m. in Miami, Florida.

Buses 15 and 16 have been selected for the installation of Electric Vehicles (EVs) within both the 57-bus and 118-bus systems due to their significant load demand and their connections to various transmission lines. This configuration provided a more realistic and complex scenario for analyzing the effects of EV charging on the power system. By placing EVs at the same buses across different systems, a fair comparison was conducted, effectively isolating the influence of the EVs on the power system. Each EV was assigned a load of 10 kW, culminating in a total load of 10,000 kW or 10 MW for 1,000 EVs. The simulation outcomes presented in Fig. (7) to Fig. (11) illustrated the optimized power loss and Voltage Deviation (VD) within the systems over time, reflecting the EV charging patterns. These results represented the minimum values achieved by addressing the Optimal Reactive Power Dispatch (ORPD) problem, which incorporated the EV charging patterns and system constraints, as opposed to the initial values prior to the ORPD resolution.

A comparative analysis of power loss and voltage deviation (VD) in the 57-bus and 118-bus systems, conducted before and after addressing the Optimal Reactive Power Dispatch (ORPD) problem, indicated notable decreases in both systems. In the 57-bus system, power loss was reduced from 2.35 MW to 1.83 MW, representing a 22.2% decrease, while VD fell from 0.034 p.u. to 0.027 p.u., demonstrating a reduction of 20.6% (Table 8).

**Table 8:** A comparative analysis of power loss and voltage deviation.

System	Power Loss (MW)	Reduction	Voltage Deviation (*p*.u.)	Reduction
57-bus	2.35 → 1.93	17.4%	0.034 → 0.027	20.6%
118-bus	4.21 → 3.53	16.2%	0.051 → 0.043	15.7%

In the 118-bus system, power loss decreased from 4.21 MW to 3.53 MW, which corresponded to a 16.2% reduction, and VD declined from 0.051 p.u. to 0.043 p.u., reflecting a 15.7% decrease. In summary, the resolution of the ORPD problem led to substantial reductions in both power loss and VD across the two systems, with the 57-bus system exhibiting more significant improvements due to its smaller scale.

## Conclusions

The rise of Electric Vehicles (EVs) has made the Optimal Reactive Power Dispatch (ORPD) more intricate. EVs have a significant impact on power grid dynamics, making it necessary to address various challenges. The primary objectives of ORPD are to maintain voltage profiles, minimize power losses, and ensure system stability. However, the integration of EVs can worsen voltage fluctuations and losses, thereby straining transmission and distribution networks. On the other hand, EVs can also function as distributed energy resources, enabling bidirectional power flow through vehicle-to-grid systems. To achieve optimal utilization without compromising user driving needs, careful coordination of charging schedules is required, taking into account battery capacity and state of charge. The ORPD problem must strike a balance between grid operational demands and the decentralized and mobile nature of EVs. In this research paper, a novel metaheuristic optimization algorithm called the Promoted Osprey Optimizer (POO) was proposed to address the ORPD problem in the presence of EVs. Inspired by the hunting behavior of ospreys, a type of prey bird, the POO algorithm offered an efficient approach to explore the search space and avoid local optima. It incorporated various strategies, including diving, soaring, and gliding. To evaluate the effectiveness of the POO algorithm, it was applied to two standard test systems, comprising the IEEE 118-bus and IEEE 57-bus systems. These systems were subjected to different scenarios of EV penetration. The experimental results demonstrated that the POO algorithm outperformed several existing metaheuristic optimization techniques, including Particle Swarm Optimization Gravitational Search Algorithm (FPSOGSA), Sine-Cosine Algorithm (SCA) algorithm, Canonical Differential Evolutionary Particle Swarm Optimizer (C-DEEPSO), Enhanced Transient Search Optimization (ETSO) in terms of convergence speed and solution quality. It effectively reduced active power loss and voltage deviation in power systems, highlighting its potential for practical implementation. Furthermore, the versatility of the POO algorithm allowed for its extension to other optimization problems in power systems and diverse domains. Moving forward, our future research endeavors will focus on investigating the impact of EV integration on additional aspects of power system operation, including stability, security, and reliability. Additionally, we aim to incorporate other factors such as renewable energy sources, load uncertainty, and network constraints into the ORPD problem with EVs.

## Data Availability

The datasets used and/or analysed during the current study available from the corresponding author on reasonable request.
